# Full color palette of fluorescent d-amino acids for *in situ* labeling of bacterial cell walls†
†Electronic supplementary information (ESI) available. See DOI: 10.1039/c7sc01800b



**DOI:** 10.1039/c7sc01800b

**Published:** 2017-07-07

**Authors:** Yen-Pang Hsu, Jonathan Rittichier, Erkin Kuru, Jacob Yablonowski, Erick Pasciak, Srinivas Tekkam, Edward Hall, Brennan Murphy, Timothy K. Lee, Ethan C. Garner, Kerwyn Casey Huang, Yves V. Brun, Michael S. VanNieuwenhze

**Affiliations:** a Department of Molecular and Cellular Biochemistry , Indiana University , Bloomington , IN 47405 , USA . Email: mvannieu@indiana.edu; b Department of Chemistry , Indiana University , Bloomington , IN 47405 , USA; c Department of Bioengineering , Stanford University , Stanford , CA 94305 , USA; d Molecular and Cellular Biology (FAS) Center for Systems Biology , Harvard University , Cambridge , Massachusetts 02138 , USA; e Department of Microbiology and Immunology , Stanford University School of Medicine , Stanford , CA 94305 , USA; f Department of Biology , Indiana University , Bloomington , IN 47405 , USA . Email: ybrun@indiana.edu

## Abstract

Fluorescent d-amino acids (FDAAs) enable efficient *in situ* labeling of peptidoglycan in diverse bacterial species. Conducted by enzymes involved in peptidoglycan biosynthesis, FDAA labeling allows specific probing of cell wall formation/remodeling activity, bacterial growth and cell morphology. Their broad application and high biocompatibility have made FDAAs an important and effective tool for studies of peptidoglycan synthesis and dynamics, which, in turn, has created a demand for the development of new FDAA probes. Here, we report the synthesis of new FDAAs, with emission wavelengths that span the entire visible spectrum. We also provide data to characterize their photochemical and physical properties, and we demonstrate their utility for visualizing peptidoglycan synthesis in Gram-negative and Gram-positive bacterial species. Finally, we show the permeability of FDAAs toward the outer-membrane of Gram-negative organisms, pinpointing the probes available for effective labeling in these species. This improved FDAA toolkit will enable numerous applications for the study of peptidoglycan biosynthesis and dynamics.

## Abstract 2

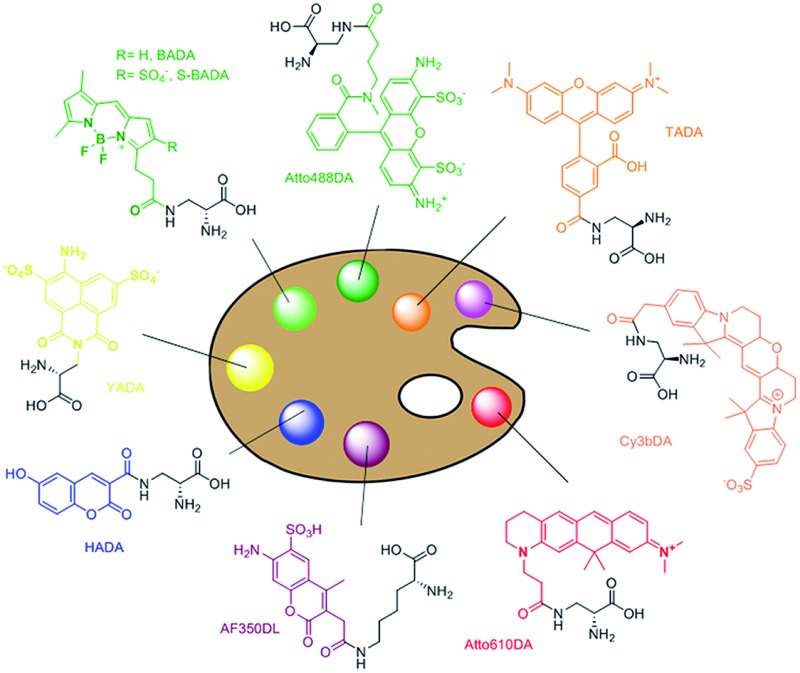
Fluorescent d-amino acids (FDAAs) enable efficient *in situ* labeling of peptidoglycan in diverse bacterial species.

## Introduction

The peptidoglycan (PG) cell wall is a macromolecular polymer of glycan strands crosslinked by short peptides that surrounds the cytoplasmic membrane of most bacteria.^1,2^ The cell wall serves as a rigid layer that protects bacteria from environmental stresses and dictates cell shape throughout their life cycle. Elucidating the mechanisms by which PG biosynthesis results in robust morphogenesis is a major challenge in microbiology, and inhibition of PG synthesis has proven a valuable strategy for the identification and development of antibiotics.

**Fig. 1:**
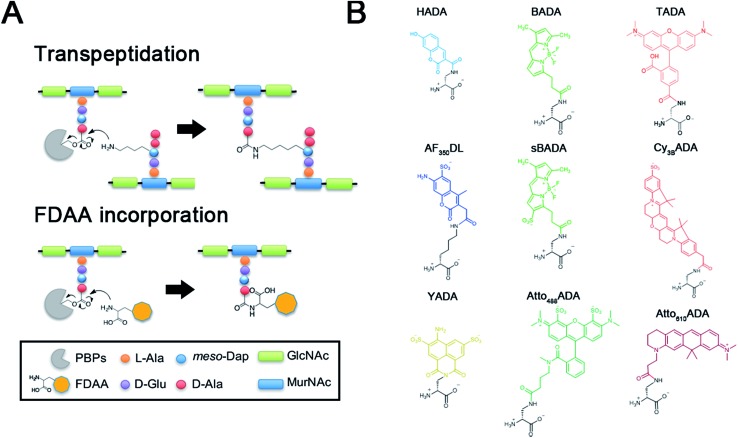
PBP-facilitated FDAA incorporation mechanism and novel FDAA structures. (A) Comparison of the transpeptidation reaction performed by penicillin-binding proteins (PBPs, top) and proposed mechanism of FDAA incorporation (bottom). The peptide-PBP intermediate (acyl donor) is attacked by the free amine of lysine in another peptide chain (acyl acceptor), which leads to cross-linking. FDAAs mimic the acyl acceptor to interact with the peptide-PBP intermediate. (B) Structures of FDAAs reported in this study.

Prior efforts directed at visualizing the sites of nascent PG growth have relied on fluorescently modified antibiotics or fluorescent lectins such as wheat germ agglutinin.^3–6^ We recently reported a new strategy that utilized a novel set of metabolic probes, fluorescent d-amino acids (FDAAs), for *in situ* PG labeling/monitoring.^7–9^ This approach relies on the inherent promiscuity of the PG biosynthetic pathway to incorporate small molecules conjugated to a d-amino acid backbone into the sites of new PG synthesis. This promiscuity is thought to be the result of a d-amino acid exchange reaction conducted by ubiquitous transpeptidases, penicillin-binding proteins (PBPs) and/or l,d-transpeptidases (Ldts) (Fig. 1A).^10–12^ The FDAA technology has demonstrated efficacy in diverse bacterial species, enabling spatiotemporal tracking of PG synthesis and modification in real-time and in live bacterial cells.^13–24^

To further enhance the utility of FDAAs in a variety of labeling applications, we report the design and synthesis of a new set of FDAA probes that cover five spectrally separable colors of the optical spectrum ranging from violet to far-red (Fig. 1B). Additionally, we provide photochemical, physical, and biological characterizations that will serve as a valuable resource when choosing the optimal FDAA for a labeling experiment of interest.

## Results and discussion

### Design and synthesis of improved FDAAs

A new FDAA should follow the basic design principles for biomolecular fluorescent probes: a “carrier” moiety to specifically bind to the target of interest (*i.e.*, a d-amino acid backbone), a fluorophore that emits light upon excitation, and a linker to join the two together.^25^ Parameters defining an ideal fluorescent probe can be largely separated into two categories: (1) photochemical properties that determine the performance of probes in microscopy, such as excitation/emission spectra and brightness, and (2) physical properties that are related to molecular interactions between probes, their targets, and their surroundings, such as solubility and stability. Optimal labeling with probes such as FDAAs relies on these properties, as well as appropriate labeling and imaging conditions required for a specific use/application.

FDAAs are biocompatible, and their labeling provides a fluorescent readout of PG synthesis and remodeling activity.^8^ Combining FDAAs with protein labels and/or fluorescent antibiotics and with super-resolution imaging technologies provides a powerful experimental approach to elucidate the spatiotemporal regulation necessary for robust bacterial growth and division.^14,24^ Sequential pulses with differently colored FDAAs enable sub-cellular tracking of PG growth *via* “virtual time-lapse” microscopy that reveals the history of PG synthesis and remodeling sites during an experiment as reported by the emission and detection of each color.^9,14^ Given the demonstrated utility of FDAA probes, we sought to expand the available FDAA color palette with probes possessing improved photochemical and/or physical properties, thereby further enhancing the utility of this valuable class of probes in various experimental applications.

**Fig. 2:**
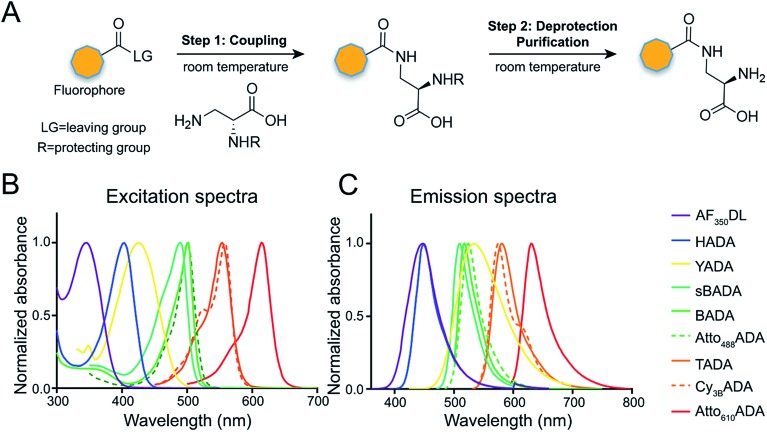
Synthesis scheme and spectra of FDAAs. (A) FDAA synthesis is achieved by coupling conjugation-activated fluorophores with 3-amino-d-alanine (3-aminopropionic acid) or d-lysine. (B and C) Excitation (B) and emission (C) spectra of FDAAs in phosphate-buffer saline (1× PBS) at pH 7.4. Absorbance and fluorescence intensity were normalized to their maximum values.

The general synthetic strategy for FDAAs is composed of three straightforward steps (Fig. 2A): (1) coupling of an N^α^-protected-d-amino acid to an activated fluorophore, purchased in its pre-activated form or synthesized in-house (Table 1), (2) removal of the N^α^ protecting group, and (3) purification of the FDAA. The coupling reaction is usually carried out with an activated fluorophore and a d-amino acid with Boc/Fmoc-protected α-amine under basic conditions and at ambient temperature. Boc-deprotection is achieved by dissolving the coupling product in a mixture of dichloromethane (DCM) and trifluoroacetic acid (TFA) at a 1 : 1 ratio and stirring at ambient temperature for approximately 2 h. An Fmoc-protecting group can be removed by suspension of the coupling product in DCM followed by treatment with 1,8-diazabicycl0(5.4.0)undec-7-ene (DBU). The crude FDAA product(s) can be purified using reverse-phase high-performance liquid chromatography (HPLC, 1 : 1 acetonitrile/water).

**Table 1:** Spectroscopic data and synthesis information of the FDAAs in the study

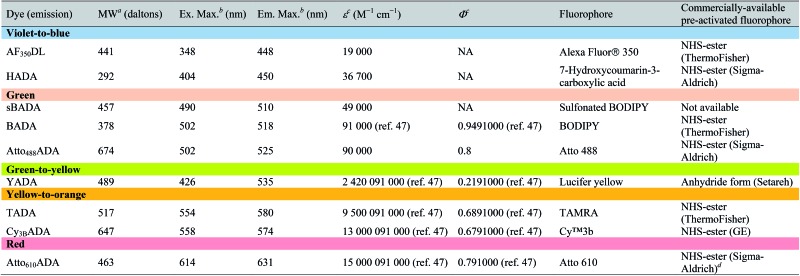

^*a*^Values for unsalted FDAAs. The molecular weight of conjugated salt was not included.

^*b*^FDAA spectra were measured in PBS buffer at 1× pH 7.4 (containing 0.1% DMSO).

^*c*^Extinction coefficient (*ε*) or quantum yield (*Φ*) of fluorophores used for FDAA synthesis. Values are from the dye manufacturer, except as noted from ref. 47. NA: not available.

^*d*^Synthesis of Atto_610_ADA using commercially available Atto_610_ NHS ester resulted in a less stable product (data not shown). We modified the structure of the linker moiety, producing stable Atto_610_ADA. Please note that the commercially available Atto_610_ NHS ester contains a butyric linker. See ESI for detailed synthesis scheme.

In an effort to develop a probe to expand FDAAs into the violet-to-blue region of the visible spectrum, we first attempted to couple an activated Alexa Flour 350 dye with 3-Amino-d-alanine. The chemical instability of the product obtained after the deprotection reaction (data not shown) prompted us to use d-lysine instead, which resulted in the stable AF_350_DL (Alexa Flour® 350 d-Lysine) (Table 2 and Fig. 2B). For green-emission probes, in addition to previously reported BADA (BODIPY-FL 3-amino-d-alanine), we further prepared sBADA (sulfonated BODIPY-FL 3-amino-d-alanine) and Atto_488_ADA (Atto 488 3-amino-d-alanine) from sulfonated BODIPY-FL and Atto 488 dye, respectively.^26,27^ For a new green-to-yellow emitting probe that would be spectrally separable from green FDAAs, we used Lucifer yellow to construct YADA (Lucifer Yellow 3-amino-d-alanine). For yellow-to-orange emission probe, in addition to TADA/TDL (TAMRA 3-amino-d-alanine/d-Lysine),^15^ we prepared Cy_3B_ADA (Cy3B 3-amino-d-alanine) from Cy™3B. Finally, we synthesized a red-to-far-red FDAA, Atto_610_ADA (Atto 610 3-amino-d-alanine), based on the reported Atto 610 dye.

**Table 2:** Photochemical and physical characterization of the FDAAs in this study

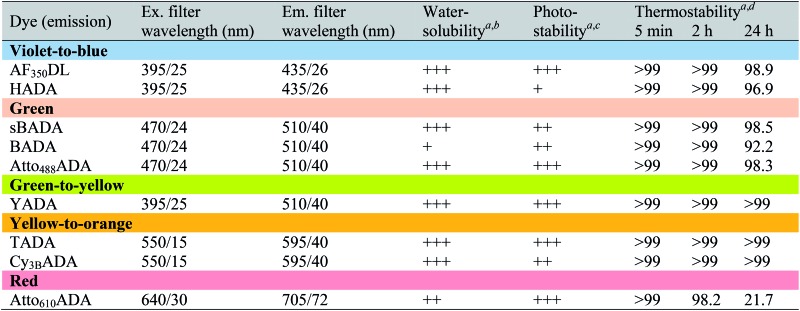

^*a*^Data were measured in PBS buffer at pH 7.4 (containing 0.1% DMSO).

^*b*^FDAAs were assigned “+++”, “++”, or “+” if the measured distribution coefficient log *D*_7.4_ was <–1, between –1 and 0, or >0, respectively.

^*c*^FDAAs were assigned “+++”, “++”, or “+” if the measured exponential decay coefficient was <0.1, 0.1–1, or >1, respectively.

^*d*^The data represent signal retention of absorbance of dye solution incubated at 37 °C for 5 min, 2 h, or 24 h, compared to the corresponding initial value.

### Photochemical properties and labeling with new FDAAs

In fluorescence microscopy, the separation of excitation and emission wavelengths of a probe is achieved by selecting filters to block or pass wavelengths specific for that probe. Under physiological conditions (in 1× phosphate-buffer saline, or PBS, at pH 7.4), AF_350_DL (maximum *λ*_ex/em_ = 348/448 nm) is ideally excited by ultraviolet light (∼350 nm). However, AF_350_DL can be partially excited by a visible violet light source (395/25 nm) and imaged using a typical blue filter set (435/26 nm, Tables 2, S3 and Fig. S1†). Thus, AF350DL can be used as an alternative of HADA (maximum *λ*_ex/em_ = 400/450 nm), a previously published blue FDAA. BADA (maximum *λ*_ex/em_ = 502/518 nm), sBADA (maximum *λ*_ex/em_ = 490/510 nm) and Atto_488_ADA (maximum *λ*_ex/em_ = 502/525 nm) can be visualized using a blue light source (470/24 nm) and a green emission filter (510/40 nm, Table 2). By comparison, YADA (maximum *λ*_ex/em_ = 426/535 nm) has a shorter excitation wavelength but a red-shifted and wider emission spectrum. The large Stokes shift and wider excitation/emission spectra of YADA enable it to be distinguished from other blue-, green-, and red-emitting FDAAs when appropriate microscopy settings are used. For example, YADA can be excited by a violet light source and detected by a green filter (or even by a red filter if necessary). Similar to the published yellow-to-orange-emitting TADA (maximum *λ*_ex/em_ = 554/580 nm), Cy_3B_ADA (maximum *λ*_ex/em_ = 558/574 nm) can be excited by green light (550/15 nm) and visualized by a red filter (595/40 nm, Table 2). Finally, Atto_610_ADA (maximum *λ*_ex/em_ = 614/631 nm) can be excited by a red light source (640/30 nm) and detected through use of a far-red filter (705/72 nm). The extinction coefficient (*ε*) and quantum yield (*Φ*) of fluorophores used for FDAA synthesis are shown in Table 1. Since no significant changes in the excitation and emission spectra were observed after these fluorophores were coupled to d-amino acids, we expect the corresponding FDAAs to retain similar extinction coefficients and quantum yields.

Two commonly utilized FDAA labeling strategies differ from each other in their respective labeling times. Long-pulse labeling experiments usually result in labeling of the whole cell, while short-pulse labeling enables visualization of the sites of transpeptidation activity during the pulse period. We confirmed the incorporation of our new FDAAs into PG by growing cells of the Gram-negative *Escherichia coli* and Gram-positive *Bacillus subtilis*, two evolutionarily distant model organisms, in the presence of these molecules for several generations (Fig. S2†). No toxic effects were observed. In addition, pre-fixed cells were not labeled, suggestive of metabolic incorporation of these FDAAs (Fig. S3†). TADA and Atto_610_ADA labeling in pre-fixed *E. coli* showed non-specific signal inside the cell (Fig. S3†). This might result from the trapped probes inside the cells after fixation which changes outer-membrane permeability toward FDAAs. Indeed, we also observed increased background intensity of FDAAs upon ethanol fixation in other Gram-negative species, such as *C. crescentus* (data not shown). Thus, control experiments using live cells were highly recommended if one needs to do cell fixation. Labeling using the l-isomer of TADA (TALA) showed no signal in live *E. coli* cells (data not shown), confirming the metabolic incorporation of the probe.

**Fig. 3:**
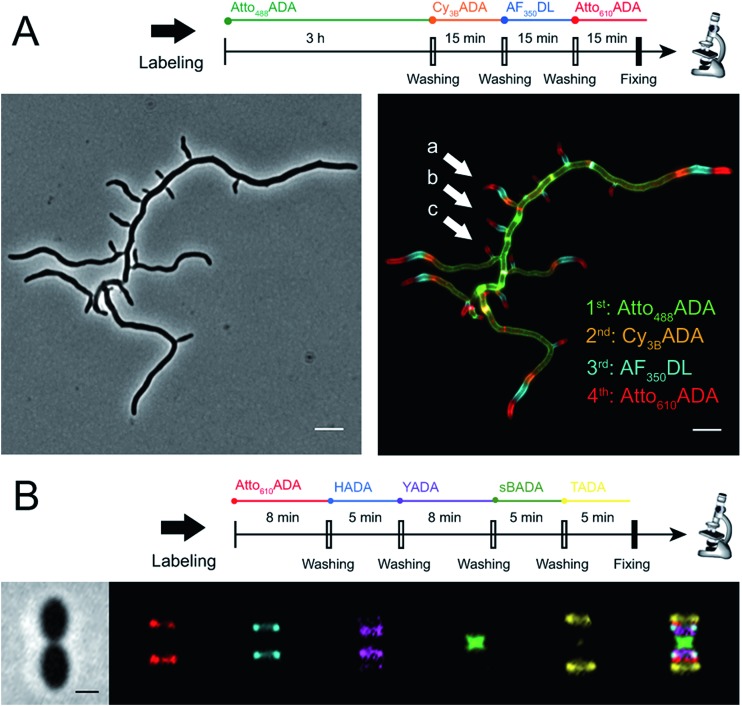
Virtual time-lapse FDAA labeling. (A) *Streptomyces venezuelae* cells were sequentially labeled with Atto_488_ADA (3 h), Cy_3B_ADA (15 min), AF_350_DL (15 min), and Atto_610_ADA (15 min), and then fixed and imaged. Left: Phase-contrast; right: fluorescence. Scale bar: 5 μm. White arrows point out new branches formed at different time points (oldest to newest: (a) to (c)). (B) *Lactococcus lactis* cells were sequentially labeled with Atto_610_ADA (8 min), HADA (5 min), YADA (8 min), sBADA (5 min), and TADA (5 min), and then fixed and imaged. Left to right: Phase channel, Atto_610_ADA, HADA, YADA, sBADA, TADA, and merged image. Scale bar: 1 μm.

The addition of more distinct colors allows for increased spatiotemporal resolution of a virtual time-lapse labeling experiment, as seen for labeling of the polar-growing and branching bacterium *Streptomyces venezuelae* with four FDAAs (Fig. 3A). *S. venezuelae* cells were successively pulsed with Atto_488_ADA (3 h), Cy_3B_ADA (15 min), AF_350_DL (15 min), and Atto_610_ADA (15 min). In this experiment, while the long-pulse labeling with Atto_488_ADA is necessary to delineate the pattern of the oldest PG at the start of the experiment, the short pulses with three distinct FDAAs resolved the polar, newer PG synthesis activity (Fig. 3A, white arrows) over a 45 min interval with a temporal resolution of 15 min per color.^9,14^ With appropriate adjustment to labeling and microscopy conditions, our expanded FDAA toolkit now enables virtual time-lapse labeling with up to five colors, in which YADA can be distinguished from blue- and green-emission probes (Fig. 3B).

### Improved physical properties of the new FDAAs

FDAAs decorate PG with high specificity that is endowed by the unique selectivity of the PG biosynthetic pathways for d-amino acids over their l-isomers. However, FDAAs with low water solubility might result in non-specific staining of the cell membrane. We determined the water solubility of all FDAAs in order to evaluate their potential for non-specific labeling. Specifically, with a given total FDAA concentration, we performed extractions of FDAAs in 1× PBS (pH 7.4) and 1-octanol and then measured the amount of FDAA distributed in each solvent layer. These measurements were used to calculate the distribution coefficient, log *D*_7.4_, where a smaller value represents greater hydrophilicity.^28,29^ Our previous experience with various probes suggest that FDAAs with log *D*_7.4_ values higher than 1 show a greater propensity for non-specific labeling. Each of the novel FDAAs reported here had log *D*_7.4_ values less than 1 (Tables 2 and S1†). Labeling experiments in pre-fixed *B. subtilis* showed no significant signal, confirming that non-specific membrane labeling by these FDAAs is minimal (Fig. S3†). The degree of non-specific labeling could also depend on the species-specific composition of membranes and experimental conditions. Of all the FDAAs, BADA has the lowest relative water solubility, which might compromise its overall utility due to non-specific labeling. This observation was the underlying motivation for preparation of the sulfonated form of BADA (sBADA), which displays significantly increased hydrophilicity. High hydrophilicity also simplifies the washing steps for cell labeling with aqueous buffers, which are required to minimize background fluorescence, and allows for use of higher FDAA concentrations in aqueous solutions (*e.g.* growth medium), which is known to enhance the signal-to-background ratio favoring the incorporation reaction.^9^

Depending on the bacterial species and the goals of the labeling experiment, the FDAA labeling duration and sample processing could range from minutes to days. Thus, in most cases, high FDAA stability during labeling at physiological conditions is desired, especially for quantitative fluorescence experiments. We examined thermal stability of the FDAAs by measuring the percentage of signal retention after an incubation in PBS (pH 7.4) at 37 °C for 5 min, 2 h, or 24 h. No significant signal reduction was observed for all the FDAAs in short incubation times between 5 min and 2 h (Table 2). After 24 h of incubation, we observed a minimal signal decrease of <10% for most of the FDAAs, suggesting that these probes exhibit high thermal stability for at least 1 day under our experimental conditions. However, Atto_610_ADA showed a significant signal reduction after 24 h of incubation, with 50% signal loss after 10 h of incubation in PBS (Fig. S4†). Thus, careful optimization and control experiments are required when using Atto_610_ADA for long-pulse labeling and quantification, especially if the experiment exceeds ∼10 h at 37 °C.

Because FDAAs are non-toxic, they can be used for time-lapse microscopy. In a typical time-lapse experiment, the high photostability of a fluorescent probe is important as the probe gets regularly exposed to the excitation light.^30–32^ To evaluate the photostability of FDAAs, we measured relative decay curves of fluorescence emission intensity for each FDAAs at their emission maximum (*λ*_em_) with continuous excitation at their corresponding excitation maximum (*λ*_ex_) in *B. subtilis* samples labeled under long-pulse conditions. Fitting to an exponential curve enabled determination of the exponential decay coefficient (EDC) as a metric of FDAA photostability, where a smaller EDC represents greater photostability (Tables 2 and S1†). Of all the FDAAs, HADA is the most sensitive to prolonged light exposure (Fig. S5†). It has a relatively high EDC value of 1.186 and a fluorescence half-life of 0.5846 s under our experimental conditions. In contrast, the other blue-to-violet FDAA, AF_350_DL, has an EDC of 0.0497 and a fluorescence half-life of 13.950 s under the same conditions. Since HADA has also shown utility for acquiring three-dimensional structured illumination microscopy (3D-SIM) images,^8,14^ which require prolonged excitation from a powerful laser (Fig. S6†), we predict all the FDAAs reported here will have sufficient photostability for most uses and applications. Furthermore, excitation-based phototoxicity could be a major issue for cell viability, especially in time-lapse microscopy experiments. Use of red-shifted TADA, Cy_3B_ADA, and Atto_610_ADA for such experiments addresses this problem, because cells are tolerant to low-energy excitation light.^31,33^ Therefore, we conclude that the new FDAAs reported here have improved photophysical properties for use in imaging applications that require prolonged excitation.

### Outer membrane permeability of FDAAs

The outer membrane (OM) of Gram-negative bacteria is an effective barrier that protects cells from toxins in the environment. Many antibiotics show low toxicity to Gram-negative species because the OM provides a permeability barrier that inhibits access to the periplasm and cytoplasm.^34^ The OM also blocks the entry of larger fluorescent probes, resulting in lower labeling efficiency. Gram-negative cells import water-soluble materials through the OM *via* porins, a class of membrane proteins that serve as transport channels. Transport is limited by the size of porin channels as well as of the cargo: a molecular weight (MW) of ∼600 g mol^–1^ was reported as the cargo cutoff size.^35,36^ The MW of FDAAs reported here ranges from ∼300 to ∼700 g mol^–1^. To investigate the OM permeability of the FDAAs in a model Gram-negative species, we labeled wild type *E. coli* BW25113 with FDAAs and measured the signal-to-background ratio (S/B) of the signal. HADA and YADA showed high labeling efficiency with S/B values >4 (Table 3). BADA, sBADA, and Atto_610_ADA had S/B values between 2 and 4. AF_350_DL, Atto_488_ADA, TADA, and Cy_3B_ADA had relatively low labeling efficiency in WT *E. coli*, with S/B values <2.

**Table 3:** The relationship between the size of an FDAA and its utility in a Gram-negative bacterium

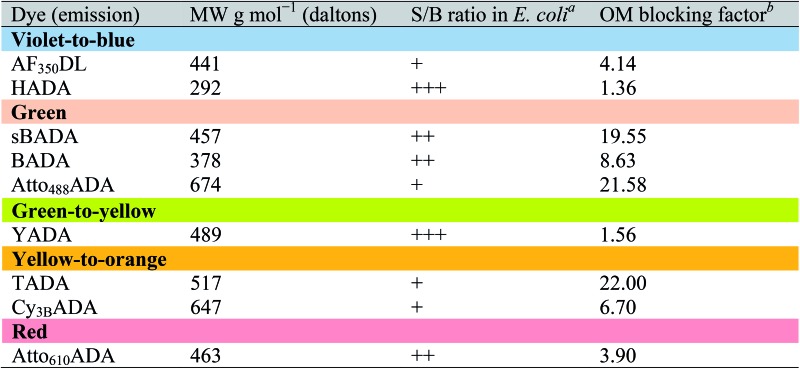

^*a*^FDAAs were assigned “+++”, ”++”, or “+” if the signal-to-background (S/B) ratio of *E. coli* BW25113 (WT) images was >4, 2–4, or <2, respectively.

^*b*^Data represent the fluorescence intensity ratio of *E. coli imp4213* BW25113 to *E. coli* BW25113 under the same labeling conditions and microscopy settings.

These S/B values are relative and are dependent upon species/strain, labeling conditions, auto-fluorescence of cells, and microscopy settings. To quantify the barrier effect of the OM on each of these FDAAs in *E. coli*, we compared FDAA intensity between wild-type *E. coli* BW25113 and *E. coli imp4213* BW25113, a mutant with increased outer membrane permeability.^37^ Under the same labeling and imaging conditions, FDAA labeling S/B values increased significantly in *E. coli imp4213* relative to the parental strain (Fig. S7A†). We represented the ratio of FDAA intensity in *E. coli imp4213* over wild-type as the “OM blocking factor” (Table 3). Consistent with displaying lower S/B values in wild-type *E. coli* relative to the other FDAAs, sBADA, TADA, and Atto_488_ADA revealed a high blocking factor of ∼20, indicating that the OM is a strong permeability barrier for each of these FDAAs. On the other hand, HADA and YADA had low blocking factors, suggesting that the OM is highly permeable to these FDAAs.

Despite the poor OM permeability to some of the FDAAs, optimization of microscope settings can effectively improve S/B of PG labeling to acceptable levels (Fig. S7B†). Moreover, the use of higher FDAA concentrations and/or minimal medium in cell labeling increases FDAA labeling efficiency. In Fig. S7C,† we show TADA and BADA labeling with different FDAA concentrations and culture medium in wild type *E. coli* BW25113. We observed a significant increase in S/B with the use of high FDAA concentrations (3 or 2 mM) compared a lower one (1 mM). An increase was also found when using M9 minimal medium compared to LB. However, additional optimization and control experiments for toxicity effects are strongly recommended when changing experimental conditions.

### FDAAs for STORM

**Fig. 4:**
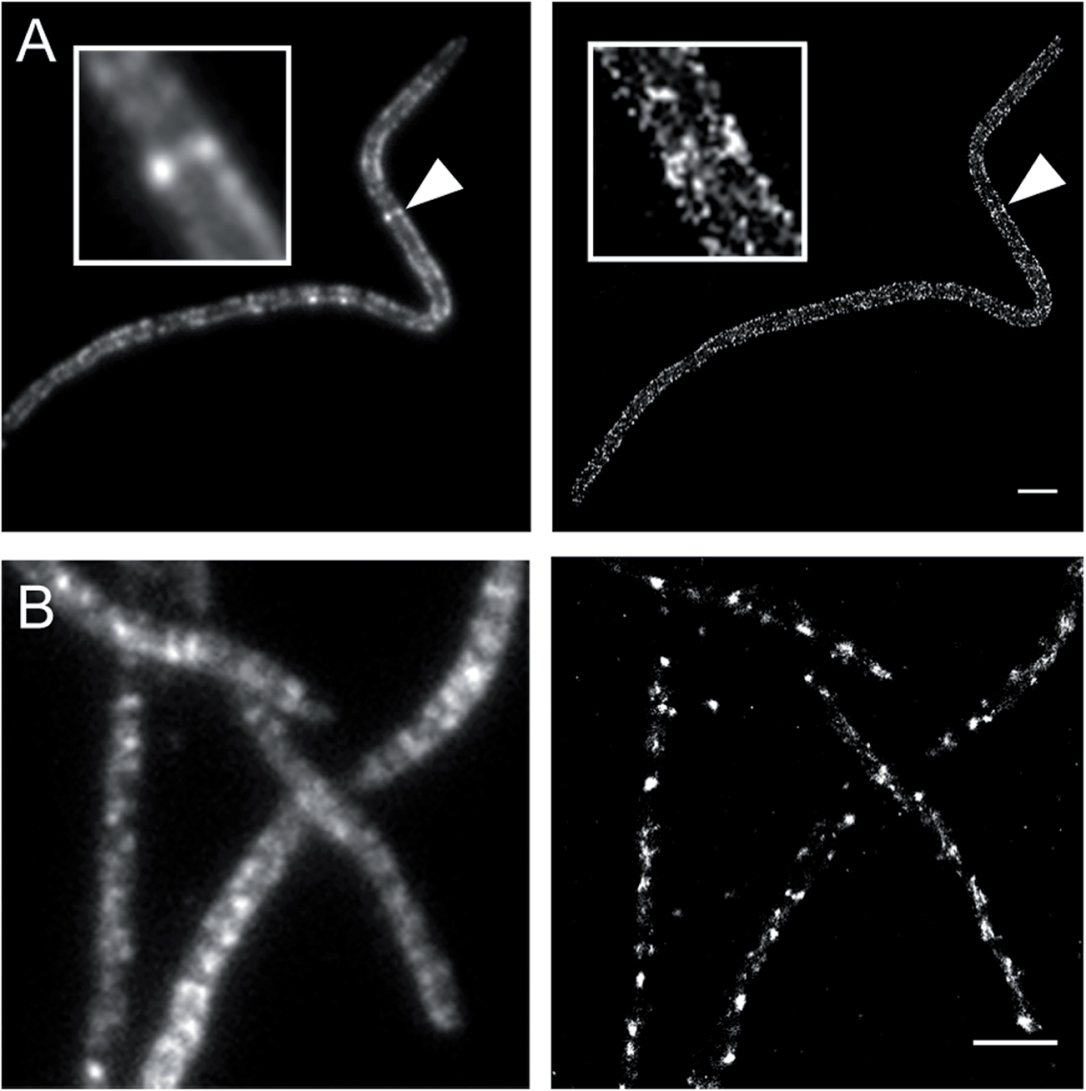
Stochastic optical reconstruction microscopy (STORM) of *E. coli imp4213* Δ*6* BW25113 cells lacking all l,d-transpeptidases. Δ*6* cells were labeled with Cy_3B_ADA (A) and TDL (B). Left: Epifluorescence microscopy; right panel: STORM (right). *E. coli imp4213* Δ6 cells were treated with cephalexin for 2 h, washed, and then pulsed with 1 mM Cy_3B_ADA/TDL for 2 min. Insets in (A) and arrow head highlight a division septum. Scale bar: 2 μm.

Currently, stochastic optical reconstruction microscopy (STORM) and related technologies offer the highest, sub-diffraction-limit, spatial resolution achievable with fluorescence microscopy.^38^ Atto 488 and Cy3B fluorophores have been reported to have outstanding applicability for direct STORM (dSTORM) and reductive caging STORM (rcSTORM), respectively.^39,40^ Therefore, Atto_488_ADA and Cy_3B_ADA should enable STORM for cell-wall imaging. Indeed, we conducted rcSTORM visualization of *E. coli* cell wall using Cy_3B_ADA and the outer-membrane permeable *imp* strain (Fig. 4A). In addition, we note that TADA/TDL also has the potential to be visualized *via* dSTORM^41^ and rcSTORM (Fig. 4B). Further optimization is highly recommended to acquire reliable reconstruction in STORM.

## Conclusion and remarks

The synthesis and characterization of the improved FDAA probes described here should be useful when choosing an optimal probe for PG labeling. The new colors yellow (YADA) and red (Atto_610_ADA) that can be spectrally separated from the typical blue (*e.g.* HADA), green (*e.g.* BADA), and orange-to-red (*e.g.* TADA) FDAAs will add versatility to the toolset, especially when pairing multiple FDAAs with fluorescent protein fusions. The increased water solubility of the new FDAAs compared to older FDAAs (*e.g.* sBADA *vs.* BADA) will improve S/B ratios of PG labeling in several ways: (1) by allowing pulses with higher FDAA concentrations; (2) by simplifying washes, and (3) by decreasing the non-specific binding to membranes. Similarly, FDAAs with improved photostability (*e.g.* AF_350_DL *vs.* HADA) or with STORM capabilities (*e.g.* Cy_3B_ADA, Atto_488_ADA and TADA) will facilitate PG imaging with increased spatial resolution when combined with super-resolution microscopy techniques.^14^

Phototoxicity is a major issue when frequent light exposure is required in an experiment such as time-lapse microscopy. Here, we also introduce Atto_610_ADA, a small, photostable FDAA that is even more red-shifted than the orange-to-red FDAA, TADA. The outer membrane permeability of Atto_610_ADA will allow tracking of PG remodeling in Gram-negative bacteria *via* time-lapse microscopy experiments in far-red region. Finally, our effort to determine the FDAA size dependency for OM permeability (OM blocking factor) not only confirms that FDAAs larger than ∼500 Da (TADA and Cy_3B_ADA) are excluded by the OM, but also reveals that other factors might be equally important as size for determining permeability. Specifically, although sBADA (MW: 457 Da) is smaller than the similarly sulfonated FDAA YADA (MW: 489 Da), sBADA is approximately 10 times less permeable than YADA. This discrepancy could result from alternative OM transport mechanisms such as lipid-mediated diffusion.^42,43^

The modular and simple design of our fluorescent amino acids allows convenient conjugation to a variety of bright and common commercially available organic probes. Such fluorescent amino acids have a variety of potential uses. Fluorescent l-amino acids have found use in peptide synthesis.^44^ More importantly, encoding brighter, red-shifted, yet still relatively small fluorescent amino acids (such as the l-enantiomer of Atto_610_ADA), into proteins could be possible with the availability of new technologies, which in turn would address current issues with utilizing fluorescent protein fusions such as size and maturation.^45^ As we have demonstrated, fluorescent d-amino acids serve as ideal PG probes to elucidate bacterial cell growth and division in high spatiotemporal resolution,^14,24^ or to simply label bacterial cell surfaces for a variety of other applications in diverse bacteria.^13,16–19,21,22,46^ We provide here an improved toolset of FDAAs to study *in situ* peptidoglycan synthesis for various purposes that range from identification of modes of cell growth, to peptidoglycan metabolism and turnover, to peptidoglycan–enzyme interactions.

## Methods

See compiled ESI.†

## References

[cit1] Typas A., Banzhaf M., Gross C. A., Vollmer W. (2011). Nat. Rev. Microbiol..

[cit2] Vollmer W., Blanot D., de Pedro M. A. (2008). FEMS Microbiol. Rev..

[cit3] Daniel R. A., Errington J. (2003). Cell.

[cit4] Tiyanont K., Doan T., Lazarus M. B., Fang X., Rudner D. Z., Walker S. (2006). Proc. Natl. Acad. Sci. U. S. A..

[cit5] Ursell T. S., Nguyen J., Monds R. D., Colavin A., Billings G., Ouzounov N., Gitai Z., Shaevitz J. W., Huang K. C. (2014). Proc. Natl. Acad. Sci. U. S. A..

[cit6] Kocaoglu O., Calvo R. A., Sham L.-T., Cozy L. M., Lanning B. R., Francis S., Winkler M. E., Kearns D. B., Carlson E. E. (2012). ACS Chem. Biol..

[cit7] Hsu Y. P., Meng X. and VanNieuwenhze M. S., in Methods in Microbiology, ed. H. Colin and J. J. Grant, Academic Press, 2016, vol. 43, pp. 3–48.

[cit8] Kuru E., Hughes H. V., Brown P. J., Hall E., Tekkam S., Cava F., de Pedro M. A., Brun Y. V., VanNieuwenhze M. S. (2012). Angew. Chem., Int. Ed..

[cit9] Kuru E., Tekkam S., Hall E., Brun Y. V., Van Nieuwenhze M. S. (2015). Nat. Protoc..

[cit10] Lupoli T. J., Tsukamoto H., Doud E. H., Wang T.-S. A., Walker S., Kahne D. (2011). J. Am. Chem. Soc..

[cit11] Qiao Y., Lebar M. D., Schirner K., Schaefer K., Tsukamoto H., Kahne D., Walker S. (2014). J. Am. Chem. Soc..

[cit12] Cava F., de Pedro M. A., Lam H., Davis B. M., Waldor M. K. (2011). EMBO J..

[cit13] Bartlett T. M., Bratton B. P., Duvshani A., Miguel A., Sheng Y., Martin N. R., Nguyen J. P., Persat A., Desmarais S. M., VanNieuwenhze M. S., Huang K. C., Zhu J., Shaevitz J. W., Gitai Z. (2017). Cell.

[cit14] Bisson Filho A. W., Hsu Y.-P., Squyres G., Kuru E., Wu F., Jukes C., Sun Y., Dekker C., Holden S., VanNieuwenhze M., Brun Y., Garner E. (2017). Science.

[cit15] Boersma M. J., Kuru E., Rittichier J. T., VanNieuwenhze M. S., Brun Y. V., Winkler M. E. (2015). J. Bacteriol..

[cit16] Cserti E., Rosskopf S., Chang Y. W., Eisheuer S., Selter L., Shi J., Regh C., Koert U., Jensen G. J., Thanbichler M. (2017). Mol. Microbiol..

[cit17] Fenton A. K., Mortaji L. E., Lau D. T., Rudner D. Z., Bernhardt T. G. (2016). Nat. Microbiol..

[cit18] Fleurie A., Lesterlin C., Manuse S., Zhao C., Cluzel C., Lavergne J. P., Franz-Wachtel M., Macek B., Combet C., Kuru E., VanNieuwenhze M. S., Brun Y. V., Sherratt D., Grangeasse C. (2014). Nature.

[cit19] Lebar M. D., May J. M., Meeske A. J., Leiman S. A., Lupoli T. J., Tsukamoto H., Losick R., Rudner D. Z., Walker S., Kahne D. (2014). J. Am. Chem. Soc..

[cit20] Liechti G., Kuru E., Packiam M., Hsu Y.-P., Tekkam S., Hall E., Rittichier J. T., VanNieuwenhze M., Brun Y. V., Maurelli A. T. (2016). PLoS Pathog..

[cit21] Monteiro J. M., Fernandes P. B., Vaz F., Pereira A. R., Tavares A. C., Ferreira M. T., Pereira P. M., Veiga H., Kuru E., VanNieuwenhze M. S., Brun Y. V., Filipe S. R., Pinho M. G. (2015). Nat. Commun..

[cit22] Morales Angeles D., Liu Y., Hartman A. M., Borisova M., de Sousa Borges A., de Kok N., Beilharz K., Veening J. W., Mayer C., Hirsch A. K., Scheffers D. J. (2017). Mol. Microbiol..

[cit23] Pilhofer M., Aistleitner K., Biboy J., Gray J., Kuru E., Hall E., Brun Y. V., VanNieuwenhze M. S., Vollmer W., Horn M., Jensen G. J. (2013). Nat. Commun..

[cit24] Yang X., Lyu Z., Miguel A., McQuillen R., Huang K. K. C., Xiao J. (2017). Science.

[cit25] Zhou J., Ma H. (2016). Chem. Sci..

[cit26] Li L., Han J., Nguyen B., Burgess K. (2008). J. Org. Chem..

[cit27] Loudet A., Burgess K. (2007). Chem. Rev..

[cit28] Sangster J. (1989). J. Phys. Chem. Ref. Data.

[cit29] Scherrer R. A., Howard S. M. (1977). J. Med. Chem..

[cit30] Bernas T., Zarebski M., Dobrucki J. W., Cook P. R. (2004). J. Microsc..

[cit31] Hoebe R. A., Van Oven C. H., Gadella T. W. J., Dhonukshe P. B., Van Noorden C. J. F., Manders E. M. M. (2007). Nat. Biotechnol..

[cit32] Song L., van Gijlswijk R. P., Young I. T., Tanke H. J. (1997). Cytometry.

[cit33] Dixit R., Cyr R. (2003). Plant J. Cell Mol. Biol..

[cit34] Delcour A. H. (2009). Biochim. Biophys. Acta, Proteins Proteomics.

[cit35] Nikaido H., Vaara M. (1985). Microbiol. Rev..

[cit36] Yoshimura F., Nikaido H. (1985). Antimicrob. Agents Chemother..

[cit37] Sampson B. A., Misra R., Benson S. A. (1989). Genetics.

[cit38] Rust M. J., Bates M., Zhuang X. (2006). Nat. Methods.

[cit39] Dempsey G. T., Vaughan J. C., Chen K. H., Bates M., Zhuang X. (2011). Nat. Methods.

[cit40] Vaughan J. C., Jia S., Zhuang X. (2012). Nat. Methods.

[cit41] Klein T., Loschberger A., Proppert S., Wolter S., van de Linde S., Sauer M. (2011). Nat. Methods.

[cit42] Alberts B., Johnson A. and Lewis J., in Molecular Biology of the Cell, Garland Science, New York, 4th edn, 2002.

[cit43] Camenisch G., Alsenz J., van de Waterbeemd H., Folkers G. (1998). Eur. J. Pharm. Sci..

[cit44] Harkiss A. H., Sutherland A. (2016). Org. Biomol. Chem..

[cit45] Luo J., Uprety R., Naro Y., Chou C., Nguyen D. P., Chin J. W., Deiters A. (2014). J. Am. Chem. Soc..

[cit46] Fura J. M., Kearns D., Pires M. M. (2015). J. Biol. Chem..

[cit47] McNamara G., McNamara 2007 Fluorophore Data Tables, Online Source, 2007.

