# Supplementary material for: Full color palette of fluorescent d-amino acids for in situ labeling of bacterial cell walls
Source: Chem Sci. 2017 Jul 7;8(9):6313–21. doi: 10.1039/c7sc01800b (PMC5628581; doi:10.1039/c7sc01800b)
Supplement: Supplementary file 1 [file SC-008-C7SC01800B-s001.pdf]

Electronic Supplementary Material (ESI) for Chemical Science.
This journal is © The Royal Society of Chemistry 2017

Supporting Information for

Full color palette of fluorescent D-amino acids for *in situ* labeling of bacterial cell walls

Yen-Pang Hsu*, Jonathan Rittichier*, Erkin Kuru*, Jacob Yablonowski, Erick Pasciak, Srinivas Tekkam, Edward Hall, Brennan Murphy, Timothy K. Lee, Ethan C. Garner, Kerwyn Casey Huang, Yves V. Brun†, and Michael S. VanNieuwenhze†

† Corresponding author

*Contributed equally

Correspondence to: mvannieu@indiana.edu. ybrun@indiana.edu

Table of Content

SI- FDAA synthesis........ 2

SI- Methods of data acquisition........ 11

SI- Figures........ 16

SI- Tables........ 31

SI- References........ 34

For Atto$_{488}$ADA synthesis (Fig. S8)

6-amino-9-(2-((4-((2-amino-2-carboxyethyl)amino)-4-oxobutyl)(methyl)carbamoyl)phenyl)-4,5-disulfo-3H-xanthen-3-iminium

To a stirring solution of **Atto 488 NHS ester** (Atto 488 NHS, 5 mg, 0.0073 mmol) in DMF (anhydrous, 0.107 mL) was added Boc-D-Dap (1.5 mg, 0.0073 mmol, 1 equiv). DIEA (1.34 µL) was then added via a micropipette. The reaction mixture was stirred at room temperature for 16 h. The reaction mixture was concentrated *in vacuo* and the crude residue was treated with TFA:DCM (10 mL of 1:1) for 2 h. The crude product was concentrated *in vacuo,* purified via reverse-phase HPLC (10–90% acetonitrile over 50 min), and lyophilized to yield the desired product as a dark red solid (1.3 mg, 23% yield, 0.0016 mmol, TFA salt). Apparent inseparable isomers (~3:1), major isomer reported: ^{1}H NMR (600 MHz, Deuterium Oxide) δ 7.83 – 7.77 (m, 2H), 7.68 (d, *J* = 6.8 Hz, 1H), 7.54 (d, *J* = 7.2 Hz, 1H), 7.31 (dd, *J* = 9.3, 4.6 Hz, 2H), 7.01 (dd, *J* = 9.6, 2.5 Hz, 2H), 3.88 (m, 1H), 3.76 (dd, *J* = 14.8, 3.3 Hz, 1H), 3.56 (dd, *J* = 14.9, 7.9 Hz, 1H), 3.30-3.22 (m, 2H), 2.93 (s, 1H), 2.88 (s, 2H), 2.64 (s, 1H), 2.19 (t, *J* = 7.1 Hz, 0H), 2.09 (d, *J* = 9.2 Hz, 0H), 1.96 (t, *J* = 7.4 Hz, 0H), 1.65 (t, *J* = 7.9 Hz, 2H), 1.51 – 1.38 (m, 2H), 1.23 (s, 0H), 1.19 (s, 0H). TOF-MS (ES+) *m/z* [M+H]+ calculated for $C_{28}H_{30}N_5O_{11}S_2$ (M+H) 676.1378; found 676.1383.

For AF$_{350}$DL synthesis (Fig. S9)

N6-(2-(7-amino-4-methyl-2-oxo-6-sulfo-2H-chromen-3-yl)acetyl)-D-lysine

To a stirring solution of **Alexa Flour 350 NHS ester** (AF 350 NHS, 5 mg, 0.012 mmol) in DMF (anhydrous, 0.12 mL) was added Boc-D-Lys (4.2 mg, 0.017 mmol, 1.4 equiv). DIEA (10 µL) was then added via a micropipette. The reaction mixture was stirred at room temperature for 16 h. The reaction mixture was concentrated *in vacuo* and the crude residue was then stirred in TFA:DCM (10 mL of 1:1) for 2 h. The crude product was concentrated *in vacuo,* purified via reverse-phase HPLC (10–50% acetonitrile over 20 min), and lyophilized to yield the desired product as a white solid (3.2

mg, 39% yield, 0.0047 mmol, DIEA/TFA salt). 1H NMR (400 MHz, Deuterium Oxide) δ 8.05 (s, 1H), 6.74 (s, 1H), 4.01 (t, J = 6.3 Hz, 1H), 3.73 (dd, J = 7.8, 5.4 Hz, 1H), 3.59 (s, 2H), 3.23 (t, J = 7.1 Hz, 3H), 2.40 (s, 3H), 2.06 – 1.84 (m, 1H), 1.63 – 1.52 (m, 2H), 1.50 – 1.39 (m, 1H). TOF-MS (ES+) *m/z* $[M+H]^+$ calculated for $C_{18}H_{24}O_8N_3S_1$ (M+H) 442.1279; found 442.1275.

For Atto$_{610}$ADA synthesis (Fig. S10)

Methyl 3-(6-formyl-3,4-dihydroquinolin-1(2*H*)-yl)propanoate (Atto 610-P1):

The following procedure was adapted from previously published literature (1). To a stirring solution of substrate (19.68 g, 89.8 mmol) in a DCM/DMF mixture (28.0 mL DMF, ~150 mL DCM) at 0 °C was slowly added $POCl_3$ (10.0 mL, 107.3 mmol). The reaction was allowed to warm to room temperature. The reaction was quenched with cold water followed by the addition of $NaHCO_3$ (saturated). The resulting crude mixture was extracted with EtOAc and washed with brine, dried over sodium sulfate, and concentrated *in vacuo.* The resulting crude product was then separated by column chromatography (1:4 EtOAc/Hexane) to provide an off-white solid (**Atto 610-P1,** 6.16 grams, 24.9 mmol, 28% yield). ^{1}H NMR (500 MHz, Chloroform-d) δ 9.67 (s, 1H), 7.55 (dd, J = 8.6, 2.0 Hz, 1H), 7.46 (dd, J = 2.1, 1.0 Hz, 1H), 6.60 (d, J = 8.6 Hz, 1H), 3.71-3.698 (m, 5H), 3.40 (dd, J = 6.6, 4.8 Hz, 3H), 2.77 (t, J = 6.3 Hz, 3H), 2.64 (t, J = 7.2 Hz, 3H), 2.06 – 1.82 (m, 3H), -0.00 (s, 3H). TOF-MS (ES+) *m/z* (M^+) calculated for $C_{14}H_{17}NO_3$ $[M]^+$ 247.1208, found: 248.1279.

Methyl 3-(6-(hydroxymethyl)-3,4-dihydroquinolin-1(2*H*)-yl)propanoate (Atto 610-P2):

To a stirring solution of **Atto 610-P1** (4.34 g, 17.6 mmol) in MeOH (100 mL) at room temperature was added a tablet of $NaBH_4$ (1 g, 26.4 mmol). After 35 min, an additional tablet of $NaBH_4$ (1 g, 26.4 mmol) was added. After an additional 45 min, the reaction was quenched by the addition of acetone. The crude reaction mixture was then concentrated *in vacuo.* The resulting crude mixture was diluted

in brine and EtOAc. The product was extracted with EtOAc and washed with brine. The organic layer was dried over sodium sulfate and concentrated *in vacuo.* The resulting pink oil product (**Atto 610-P2**), which degrades quickly, was carried onto the next reaction without further purification (3.96 g, 15.9 mmol, 91% yield).

Atto610 Methyl Propanoate (Atto 610-P3):

To a stirring solution of **Atto 610-P2** (1.97 g, 7.48 mmol) and *N,N*-dimethyl-3-(prop-1-en-2-yl)aniline (1.29 g, 8.00 mmol) in DCM (16 mL) at 0 °C was added boron trichloride. The reaction was allowed to continue at 0 °C for 1 h. The reaction was then allowed to warm to room temperature. After an additional hour of reacting, H_2SO_4 (40 mL, concentrated) was carefully added and DCM was removed *in vacuo.* This reaction was allowed to continue for an additional 2 h. A copious amount of ethanol (~500 mL) was added followed by the addition of $(nBu)_4NIO_4$ (0.8 g, 1.80 mmol). The reaction was then refluxed for 10 min. The reaction was then allowed to cool to room temperature and stir overnight. The crude mixture was then concentrated *in vacuo* and the product was extracted with several portions of DCM. The crude product was purified by reverse phase HPLC (10-90% ACN/H_2O over 20 min) to provide a blue powdery solid TFA salt (close to 1:1 mixture of ethyl ester and methyl ester**,** 0.926 g, 1.84 mmol, 25%). For the ethyl ester compound: ^{1}H NMR (600 MHz, Acetonitrile-d3) δ 7.89 (s, 1H), 7.69 – 7.53 (m, 1H), 7.30 (s, 1H), 7.11 – 6.98 (m, 1H), 6.84 (dd, J = 9.1, 2.3 Hz, 1H), 4.15 – 3.99 (m, 2H), 3.88 (t, J = 7.0 Hz, 2H), 3.56 (t, J = 5.7 Hz, 2H), 3.23 (d, J = 1.3 Hz, 6H), 2.71 (t, J = 6.3 Hz, 2H), 2.66 (dd, J = 7.6, 6.3 Hz, 2H), 1.61 (d, J = 1.3 Hz, 6H), 1.15 (m, 3H); TOF-MS (ES+) *m/z* (M^+) calculated for $C_{26}H_{33}N_2O_2$ $[M]^+$ 405.2537, found: 405.2545.

Atto610 Propanoic Acid (Atto610):

Atto 610-P3 (600.0 mg, 1.19 mmol) was dissolved in a perchloric acid solution of acetone/water (3 mL:50 mL:100 mL, acid/acetone/water) and heated overnight at 70 °C under a reflux condenser. The acetone was removed *in vacuo* and the product was extracted with DCM, dried over sodium sulfate and concentrated *in vacuo*. The crude product was purified on the reverse phase HPLC (10-90% ACN/H_2O over 15 min). The acetonitrile was removed *in vacuo* and the product was extracted in DCM, dried over sodium sulfate and concentrated *in vacuo* to provide the pure product (**Atto610**) as a purple, solid TFA salt (212.7 mg, 0.434 mmol, 36%) ^{1}H NMR (500 MHz, Acetonitrile-d3) δ 7.93 (s, 1H), 7.63 (d, J = 9.1 Hz, 1H), 7.35 (s, 1H), 7.11 (s, 1H), 7.07 (d, J = 2.2 Hz, 1H), 6.89 (dd, J = 9.1, 2.4 Hz, 1H), 3.93 (t, J = 7.0 Hz, 2H), 3.63 (t, J = 5.7 Hz, 2H), 3.28 (s, 6H), 2.81 – 2.71 (m, 4H), 1.66 (s, 6H). TOF-MS (ES+) *m/z* (M^+) calculated for $C_{24}H_{29}N_2O_2$ $[M]^+$ 377.2224, found: 377.2210.

(R)-1-carboxy-2-(3-(9-(dimethyliminio)-11,11-dimethyl-3,4,9,11-tetrahydronaphtho[2,3-g]quinolin-1(2H)-yl)propanamido)ethan-1-aminium (Atto$_{610}$ADA):

General procedure: To a stirring solution of the free acid **Atto 610**, under a blanket of argon in DMF (~0.1 M), was added CDI (1.2 equiv). The reaction was allowed to continue for 2 h. Boc-D-Dap was then added in one portion (1.2 equiv). After coupling overnight, the reaction solution was concentrated and the crude product was subjected to a TFA solution (1:1, TFA:DCM) for 2 h. Reverse HPLC purification (10–90% acetonitrile over 10 min, RT~7 min) provided purple-red solid (up to 37%, bis-TFA salt). ^{1}H NMR (500 MHz, Deuterium Oxide) δ 7.79 (s, 1H), 7.59 (d, *J* = 9.0 Hz, 1H), 7.20 (s, 1H), 7.06 – 7.01 (m, 2H), 7.00 (s, 1H), 6.89 (dd, *J* = 9.0, 2.2 Hz, 2H), 3.97 – 3.91 (m, 2H), 3.88 (dd, *J* = 6.4, 4.0 Hz, 1H), 3.72 (dd, *J* = 14.8, 4.0 Hz, 1H), 3.61 – 3.54 (m, 2H), 3.29 (s, 6H), 2.69 (dt, *J* = 13.1, 6.5 Hz, 4H), 1.95 – 1.86 (m, 1H), 1.52 (s, 6H). ^{13}C NMR (126 MHz, d_2o) δ 175.45, 172.32, 157.28, 157.02, 154.30, 153.51, 139.22, 136.74, 124.70, 121.17, 121.04, 119.75, 113.36, 111.43, 110.46, 55.45, 50.98,

48.39, 41.97, 40.72, 40.49, 33.74, 33.35, 28.49, 27.04, 21.08. TOF-MS (ES+) *m/z* (M^+) calculated for $C_{27}H_{35}N_4O_3$ $[M]^+$463.2709, found: 463.2682.

<u>For BADA synthesis (Fig. S11)</u>

(S)-2-amino-3-(3-(5,5-difluoro-7,9-dimethyl-5H-5l4,6l4-dipyrrolo[1,2-c:2',1'-f][1,3,2]diazaborinin-3-yl)propanamido)propanoic acid:

To a stirring solution of **BODIPY FL carboxylic acid** (161 mg, 0.55 mmol) in DMF (6 mL) was added CDI (101 mg, 0.63mmol, 1.2 equiv). This solution was then stirred at room temperature for 2 h. Boc-D-Dap-OH was then added (139 mg, 0.68mmol, 1.2 equiv.) and stirring continuously overnight. The Boc protected fluorophore was then extracted from the reaction solution with ethyl acetate, washed, dried over anhydrous sodium sulfate and concentrated *in vacuo*. It was then directly carried on to deprotection (TFA/DCM 1:1, ~2 h). The product was purified via reverse-phase HPLC (gradient, 50-90% MeCN/H_2O, over 10 min, buffered at 0.1% TFA). The pure fractions were concentrated *in vacuo* to yield the desired product **BADA**, as a red solid. (118 mg, 44 % yield, 0.24 mmol, TFA salt). ^{1}H NMR (400 MHz, DMSO-d_6) δ 8.23 (d, *J* = 5.9 Hz, 3H), 7.70 (s, 1H), 7.09 (d, *J* = 4.0 Hz, 1H), 6.35 (d, *J* = 4.0 Hz, 1H), 6.31 (s, 1H), 3.99 (t, *J* = 5.6 Hz, 1H), 3.62 (dt, *J* = 14.3, 5.3 Hz, 1H), 3.46 (dt, *J* = 13.9, 6.2 Hz, 1H), 3.09 (t, *J* = 7.9 Hz, 2H), 2.58 – 2.52 (m, 2H), 2.47 (s, 3H), 2.26 (s, 3H). ^{13}C NMR (101 MHz, dmso) δ 172.35, 169.25, 159.28, 157.48, 144.21, 134.48, 132.97, 128.90, 125.40, 120.32, 116.48, 62.38, 52.36, 33.55, 23.65, 14.52, 11.00. TOF-MS (ES+) *m/z* $[M+2Na^+-H^+]^+$ calculated for $C_{17}H_{21}O_3N_4B_1BF_2$ $(M-Na)^+$ 423.1395; found 423.1380.

<u>For sBADA synthesis (Fig. S12)</u>

Sodium 3-(3-ethoxy-3-oxopropyl)-5,5-difluoro-7,9-dimethyl-5H-5l4,6l4-dipyrrolo[1,2-c:2',1'-f][1,3,2]diazaborinine-2-sulfonate (sulfonated BODIPY FL ethyl ester):

Inspired by Burgess's procedure (2), DCM (anhydrous, 75 mL) was added to **BODIPY FL ethyl ester** (263 mg, 0.82 mmol) under an atmosphere of argon at -40 °C. Chlorosulfuric acid in DCM (65.5 µL/10 mL, 1.0 mmol, 1.2 equiv.) was then added dropwise over 10 min. After stirring the reaction for 1 h, an aqueous solution of sodium bicarbonate was added (135 mg, 0.16 M, 1.6 mmol, 2 equiv.). The product was extracted with water, washed with DCM and concentrated *in vacuo.* The product was purified via column chromatography (15% MeOH/DCM) to yield **sulfonated BODIPY FL ethyl ester**, as a red solid (200.6 mg, 58% yield). ^{1}H NMR (500 MHz, DMSO-*d6*); δ =7.73 (s, 1H), 7.03 (s, 1H), 6.34 (s, 1H), 4.09 (q, J = 7.08 Hz, 2H), 3.22 (m, 2H), 2.74 (m, 2H), 2.47 (s, 3H), 2.26 (s, 3H), 1.21 (t, J = 7.12 Hz, 3H). ^{13}C NMR (125 MHz, DMSO-*d6*); δ =172.0, 160.5, 152.6, 144.9, 138.4, 135.1, 129.7, 126.1, 126.0, 120.8, 60.0, 32.7, 22.4, 14.6, 14.1, 11.1. TOF-MS (ES+) *m/z* $[M+Na]^+$ calculated for $C_{16}H_{18}O_5N_2B_1F_2S$ $(M-Na)^+$ 399.0998; found 399.0981.

sodium 3-(2-carboxyethyl)-5,5-difluoro-7,9-dimethyl-5H-5l4,6l4-dipyrrolo[1,2-c:2',1'-f][1,3,2]diazaborinine-2-sulfonate (sulfonated BODIPY FL):

An acidic solution (THF/$H_2$0/HCl_{conc}, 12:8:4.6, 24.6mL) was added to **sulfonated BODIPY FL ethyl ester** (153 mg, 0.36 mmol). After stirring the reaction overnight, the crude mixture was concentrated *in vacuo*. The product was purified by reverse-phase HPLC (10–90% acetonitrile over 10 min), and lyophilized to yield the desired product as a red solid (61.6 mg, 43% yield). ^{1}H NMR (500 MHz, DMSO-*d6*); δ = 7.71 (s, 1H), 7.04 (s, 1H), 6.33 (s, 1H), 3.19 (m, 2H), 2.67 (m, 2H), 2.47 (s, 3H), 2.26 (s, 3H). ^{13}C NMR (125 MHz, DMSO-*d6*); δ = 173.6, 160.5, 153.2, 144.8, 138.2, 135.1, 129.7, 126.2, 126.0, 120.8, 32.9, 22.6, 14.6, 11.1. TOF-MS (ES+) *m/z* $[M]^-$ calculated for $C_{16}H_{18}O_5N_2B_1F_2S$ $[M]^-$, found 371.0692.

sodium (S)-3-(3-((2-amino-2-carboxyethyl)amino)-3-oxopropyl)-5,5-difluoro-7,9-dimethyl-5*H*-5λ^4,6 λ^4-dipyrrolo[1,2-*c*:2',1'-*f*][1,3,2]diazaborinine-2-sulfonate (sBADA)

To a stirring solution of **sulfonated BODIPY FL** (109.7 mg, 0.28 mmol) in DMF (anhydrous, 3 mL) was added CDI (55.5 mg, 0.34 mmol, 1.2 equiv.). After 2 h of acid activation, Boc-D-Dap was added in one portion (68.6 mg, 0.34 mmol, 1.2 equiv.) and the reaction continued overnight. The crude residue was then stirred in TFA:DCM (10 mL of 1:1) for 1 h at room temperature. The crude product was concentrated *in vacuo,* purified by reverse-phase HPLC (10–90% acetonitrile over 10 min) and lyophilized to yield the desired product as a red solid (50.8 mg, 38% yield, TFA salt). ^{1}H NMR (500 MHz, DMSO-*d6*); δ = 8.25, (s br, 3H), 8.05 (t, *J* = 5.9, 1H), 7.72 (s, 1H), 7.06 (s, 1H), 6.33, (s, 1H), 3.99 (dd, *J* = 6.5, 4.4, 1H), 3.55 (m, 2H), 3.22 (t, *J* = 6.4, 2H), 3.17 (s, 1H), 2.62 (t, *J* = 8.4, 2H), 2.47 (s, 3H), 2.26 (s, 3H). ^{13}C NMR (125 MHz, DMSO-*d6*); δ = 172.9, 169.3, 160.1, 154.1, 144.5, 138.2, 134.9, 129.7, 126.5, 125.9, 120.6, 52.5, 48.6, 34.1, 22.7, 14.6, 11.0. TOF-MS (ES+) *m/z* $[M+H]^{+}$ calculated for $C_{17}H_{21}O_{6}N_{4}B_{1}F_{2}SNa$ (M+H) 481.1141; found 481.1134.

For Cy_{3B}ADA synthesis (Fig. S13)

To a stirring solution of **Cy3B NHS ester** (Cy3B NHS, 10.7mg, 0.0139 mmol) in DMF (anhydrous, 0.56mL) was added Boc-D-Dap (5.7 mg, 0.0277 mmol, 2 equiv). DIEA (7.2 µL) was then added via a micropipette. The reaction mixture was stirred at room temperature for 15.5 h. The reaction mixture was concentrated *in vacuo* and the crude residue was treated with TFA:DCM (10 mL of 1:1) for 2 h. The crude product was concentrated *in vacuo,* purified via reverse-phase HPLC (10–90% acetonitrile over 15 min), and lyophilized to yield the desired product as a dark purple solid (3.6 mg, 29% yield, 0.004 mmol, DIEA/TFA salt). 1H NMR (600 MHz, DMSO-d6) δ 8.35 (t, J = 6.0 Hz, 1H), 7.99 (s, 1H), 7.80 (s, 1H), 7.68 (d, J = 8.2 Hz, 1H), 7.48 (s, 1H), 7.31 (s, 3H), 7.30 (s, 0H), 4.64 (dd, J = 11.7, 5.1 Hz, 2H), 4.32 (d, J = 13.8 Hz, 2H), 3.90 (t, J = 13.4 Hz, 2H), 3.47 – 3.41 (m, 1H), 2.05 – 1.88 (m, 2H), 1.74 – 1.68 (m, 11H), 1.35 – 1.19 (m, 2H). TOF-MS (ES+) *m/z* $[M+H^{+}]^{+}$ calculated for $C_{34}H_{39}O_{7}N_{4}S$ $(M)^{+}$ 647.2534; found 647.2515.

For YADA synthesis (Fig. S14)

Potassium 6-amino-2-(2-amino-2-carboxyethyl)-1,3-dioxo-2,3-dihydro-1*H*-benzo[de]isoquinoline-5,8-disulfonate

Lucifer yellow anhydride (100 mg, 0.22 mmol), Fmoc-*d*-Dap (215 mg, 0.66 mmol, 3 eq.), and lithium acetate buffer (20 mL, 1M, pH 5) were added to a 100 mL round bottle. This solution was stirred at refluxed for 6 h, followed by the addition of an aqueous solution of potassium (10 mL, 3.35 M) of to form a precipitate. The precipitate was filtered, washed two times with cold ethanol, and allowed to dry. The powder was then dissolved in 5 mL of DMF followed by the addition of DBU (69 µL, 0.46 mmol, 2.1 eq) and stirred at room temperature for 1 h. The solvent was removed *in vacuo* and the product was purified via reverse-phase HPLC (0–90% acetonitrile over 30 min, RT~7 min) The pure fractions were lyophilized to yield the desired product as a yellow solid as a TFA salt (70.3 mg, 56% yield). ^{1}H NMR (500 MHz, DMSO-d_6) δ 8.96 (d, *J* = 1.5 Hz, 1H), 8.67 (d, *J* = 1.5 Hz, 1H), 8.62 (s, 1H), 8.35 (d, *J* = 5.6 Hz, 3H), 4.54 (dd, *J* = 13.6, 6.6 Hz, 1H), 4.45 (dd, *J* = 13.7, 7.5 Hz, 1H), 4.18 (q, *J* = 6.4 Hz, 1H), 3.17 (s, 2H). ^{13}C NMR (126 MHz, dmso) δ 169.03, 164.18, 163.25, 149.08, 144.85, 133.08, 129.56, 129.18, 126.29, 125.27, 121.37, 119.96, 106.13, 50.53, 48.61.; TOF-MS (ES+) *m/z* $[M+H]^+$ calculated for $C_{15}H_{12}O_{10}N_3S_2$ (M+H) 457.9964; found 457.9976.

For TADA synthesis (Fig. S15)

(S)-N-(9-(4-((2-amino-2-carboxyethyl)carbamoyl)-2-carboxyphenyl)-6-(dimethylamino)-3H-xanthen-3-ylidene)-N-methylmethanaminium

To a stirring solution of 5-Carboxytetramethylrhodamine **TAMRA** (10 mg, 0.0232 mmol) in DMF (anhydrous, 1 mL) was added CDI (4.5 mg, 0.0278 mmol, 1.2 equiv). This solution was then stirred at room temperature for 2 h. Boc-D-Dap-OH for TADA was then added (0.0278 mmol, 1.2 equiv.) and

stirred continuously overnight. The reaction mixture was concentrated *in vacuo* and the crude residue was then stirred in TFA:DCM (10 mL of 1:1) for 2 h. The crude product was concentrated *in vacuo,* purified via reverse-phase HPLC (10–90% acetonitrile over 10 min), and lyophilized to yield the desired product as a red solid (4.7 mg, 27% yield, 0.0063 mmol, bis-TFA salt). 1H NMR (600 MHz, Methanol-d4) δ 8.82 (s, 1H), 8.29 (d, J = 8.1 Hz, 1H), 7.54 (d, J = 7.9 Hz, 1H), 7.12 (d, J = 9.3 Hz, 3H), 7.05 (dd, J = 9.5, 2.4 Hz, 3H), 6.99 (s, 3H), 4.26 (t, J = 6.1, 4.4 Hz, 2H), 4.07 (dd, J = 14.5, 4.1 Hz, 2H), 3.93 (dd, J = 14.5, 6.4 Hz, 2H), 3.30 (s, 36H). TOF-MS (ES+) *m/z* $[M+H]^+$ calculated for $C_{28}H_{29}O_6N_4^+$ (M+H) 517.2087; found 517.2101. The synthesis protocol of TDL has been reported in previous literature (3).

SI-Methods of data acquisition

Culture growth. Strain characteristics and growth conditions are described in Table S2. Bacterial cells were streaked from -80 °C freezer stocks (LB broth with 10% DMSO) onto LB agar plates, followed by overnight growth at 37 °C for *Escherichia coli* and *Bacillus subtilis* or 30 °C for *Streptomyces venezuelae* and *Lactococcus lactis*. Single colonies from the overnight plate were transferred to liquid LB broth and incubated in an Innova® 44R shaker with a shaking speed of 200 rpm. After cell cultures reached OD_{600} ~ 0.5, they were diluted with culture media (10x) and grown one more round until OD_{600} ~ 0.5. The cells were then used for FDAA labeling.

Fluorescent D-amino acid labeling. FDAA stock solutions were prepared in DMSO (Sigma-Aldrich ReagentPlus >99.5%) in 100 mM and stored at -20 °C before use. For long-pulse labeling in Figs. S2,S3 and Figs. S6,S7, exponential phase cells were diluted with fresh LB broth containing 0.5 or 1 mM FDAA and grown at 37 °C for 1 h. The cells were then fixed by treating with 70% ice-cold ethanol, followed by incubating on ice-bath for 1 h. The fixed cells were then washed twice with 4 °C 1x PBS (NaCl 8 g/L, KCl 0.2 g/L, Na_2HPO_4-$2H_2O$ 1.78 g/L, KH_2PO_4 0.27 g/L, pH 7.4) via centrifuge (10,000*g*, 4 min). The cell pellets were then re-suspended in 4 °C PBS, and stored on ice-bath before imaging. All ethanol-fixed cells were imaged no later than three hours after fixation. For FDAA labeling in minimal medium in Fig. S7C, exponentially growing cells from LB medium were diluted with M9 medium (500X) and incubated overnight in a 37 °C shaker. The overnight culture was diluted with fresh M9 again to OD_{600}~0.05. The cells were allowed to grow to OD_{600}~0.5 and then used for FDAA labeling.

For virtual time-lapse labeling in Fig. 3, *S. venezuelae* cells in exponential growth were diluted in fresh LB broth containing 0.25 mM $Atto_{488}$ADA and grown at 30 °C for 3 h. Cells were washed twice with pre-warmed fresh LB broth (30 °C) and collected via centrifuge (7,000 *g*, 2 min). Cells were re-suspended in fresh LB broth (30 °C) containing 0.5 mM Cy_{3B}ADA and grown for 15 min. The process was repeated for the third (AF_{350}DL, 0.5 mM) and fourth ($Atto_{610}$ADA, 0.25 mM) labeling. Labeled

cells were treated with 70% ice-cold ethanol and incubated on ice-bath for 1 h. The fixed cells were then washed twice with 4 °C 1x PBS via centrifuge and re-suspended in 4 °C 1x PBS before imaging. For virtual time-lapse labeling with *Lactococcus lactis*, cells were treated with FDAAs using the same approach as described above. $Atto_{610}$ADA: 1mM, 8 min; HADA: 2mM, 5 min; YADA: 2mM, 8 min; sBADA: 1mM, 5 min; TADA: 1mM, 5 min. Cells were collected and washed via centrifuge (9,000*g*, 2 min). YADA signal was recorded using a 595/40 emission filter with 395-nm excitation light.

For Cy_{3B}ADA and TDL STORM images in Fig. 4, a reductive caging STORM protocol optimized for bacterial cell labeling was used (4). To exponentially growing *E. coli imp4213 Δ6 Ldt* cells grown in LB broth, 35 μg/ml cephalexin was added and the cultures were incubated for 2 h; cephalexin inhibits cell division and results in elongated cells. These cells were then pulsed with 1 mM Cy_{3B}ADA/TDL for 15 s–2 min and were immediately fixed by adding ice-cold EtOH to a final concentration of 70% and incubated on ice for 15 min. Cells were then transferred into a 1.7-mL tube and the cells were washed with ice-cold H_2O twice (5 min, 9,000*g*). The cell pellet was resuspended in 300 μL freshly prepared $NaBH_4$ (1 mg/mL, in H_2O) and incubated in the dark at room temperature, leaving the cap of the tube open as H_2 gas formed. After 15 min, the redox reaction was quenched by adding 1 mL 10x PBS and the cell suspension was further incubated for 5 min at room temperature. The cells were then pelleted one final time, the cell pellet was resuspended in 5–10 μL 10x PBS, and 0.7 μL cells were spotted onto plasma-cleaned, poly-L-lysine coated coverslips and mounted with 2 μL Prolong Gold Antifade reagent. Reductive caging STORM imaging was performed within 30 min, as the reduced (i.e. dark) Cy_{3B}ADA and TDL can spontaneously re-oxidize over time.

Image sample preparation. 24x50 mm coverslips (#1.5) were used as imaging supports for the inverted microscope system. For fluorescence imaging, the slides were rinsed with ethanol and water twice, and air-dried before use. For STORM imaging, coverslips were plasma-cleaned. Cell samples were loaded onto the coverslips, followed by laying an 8x8 mm wide, 2-mm thick PBS-agar pad on

top of the cells. The coverslip-pad combination was placed onto a customized slide holder on microscopes with the pad facing upwards.

Image acquisition. Phase/fluorescence images were acquired using a Nikon Ti-E inverted microscope equipped with a 1.4NA Plan Apo 60X oil objective and Andor iXon X3 EMCCD camera. NIS-Elements AR software was used for image acquisition. Selection of light sources and filters for each FDAA is summarized in Tables 2 and S3. Light source power, EM gain, and exposure time were optimized for each experiment/specimen.

A quantitative assessment of the brightness of FDAA labeling is difficult because it is dependent on growth conditions/durations, dye concentrations, bacterial species/strain, imaging conditions (excitation and emission filters, exposure times, gains, etc.). To ensure a high signal-to-background ratio of FDAA labeling, careful optimization of microscopy settings is recommended, including light source power, exposure, and imaging interval. One can also use antifade reagents to minimize photo-bleaching of FDAAs.

STORM images were acquired on a microscope built with a Ti-E Eclipse stand (Nikon Instruments, Inc., Melville, NY, USA). A Plan Apo Lambda 100X DM (NA 1.45) (Nikon) objective was used to acquire phase-contrast images concurrently with fluorescence images. CUBE diode 405-nm and Sapphire OPSL 561-nm lasers (Coherent, Santa Clara, CA, USA) were combined into an optical fibre attached to the microscope stand. A weak cylindrical lens was introduced into the imaging path to capture three-dimensional localization information using the astigmatism imaging method (5). Shuttering of laser illumination was controlled with an acousto-optic tuneable filter (AA optoelectronics, Orsay, France) before the fibre coupler. Images were acquired with an iXon3+ 887 EMCCD (Andor Technology, South Windsor, CT, USA) camera, and synchronisation between components was achieved using μ-Manager 1.4 (6) with a microcontroller (Arduino, Almuñécar, Spain).

Image processing. Unless otherwise specified, all image processing was performed in FIJI. Images were cropped, rotated, and scaled without interpolation. Contrast and brightness were adjusted. Only linear adjustments were performed. Figure montage was achieved using Adobe Photoshop and Illustrator.

Fluorescence spectra and intensity acquisition. Spectra wavelengths and FDAA fluorescence intensities were measured using a SpectraMax M2 plate reader (Molecular Devices). 48/96-well plates (Falcon® Polystyrene) and cuvettes (polymethyl methacrylate) were used based on the experimental design as described in each section.

For spectra measurements, FDAA stock solutions were diluted with 1x PBS (pH 7.4) to a final concentration of 0.05-0.1 mM. Absorbance spectra were obtained by scanning the samples with excitation light ranging from 300 to 900 nm with an increment of 1 nm using disposable cuvettes. The wavelength at ~20 nm below the highest absorbance was used as the excitation wavelength for measuring excitation spectra. Excitation and emission spectra in Figs. 2 and S1 were generated using Prism.

Measurement of distribution coefficient ($LogD_{7.4}$). The protocol was modified from previous literature (7). FDAA stock solutions were diluted with 2 mL 1x PBS to a final concentration of 0.02 mM. The absorbance at maximum λ_{ex} was recorded. The solution was then extracted with 2 mL 1-octanol once. The PBS layer was collected and the absorbance was measured (cuvette) to calculate the amount of remaining FDAA. The $LogD_{7.4}$ value was calculated as

$$\text{LogD}_{\text{PBS, pH7.4}} = \log\frac{[\text{Solute}]_{1-\text{octanol}}}{[\text{Solute}]_{\text{PBS, pH7.4}}}$$

Measurement of FDAA thermostability. FDAA stock solutions were diluted with 1x PBS (pH 7.4) to a final concentration of 0.05 mM. Their absorbance at maximum λ_{ex} was measured and recorded. The solutions were then incubated at 37 °C and kept from light exposure. The absorbance was measured after 5 min, 2 h, or 24 h incubation. The thermostability was calculated as the ratio of final absorbance to initial absorbance of FDAA:

$$\text{Thermostability} = \frac{A_{\text{final}}}{A_{\text{initial}}} \times 100\%$$

The thermostability curve of Atto$_{610}$ADA in Fig. S5 was obtained via time-lapse measurement of absorbance in a plate reader (37 °C, 12 h). Atto$_{610}$ADA solution was prepared in a cuvette sealed with a plastic cap and parafilm to prevent solvent evaporation.

Measurement of FDAA photo-stability. FDAA-labeled *B. subtilis* cells were used for photo-stability measurements. Specifically, *B. subtilis* cells were labeled with 0.5 mM FDAA for 1 h as described above. FDAA intensity was recorded in time-lapse during a continuous light exposure at the appropriate excitation wavelength (Table 2) for ~30 s. The RAM function of NIS-Elements AR software was used for the measurement. Exponential decay curves were generated using Prism. Please note that the decay curve of FDAA intensity is dependent on imaging conditions (e.g. the power of light source). Thus, the photo-stability values reported here should only be used when comparing FDAAs in this study.

FDAA intensity quantification in Table 3 was accomplished using the MicrobeJ plugin in FIJI (8). Cell morphologies and boundaries were defined using the phase channel. The fluorescence intensity of the cells (*n*>100) was then measured and averaged using the plugin.

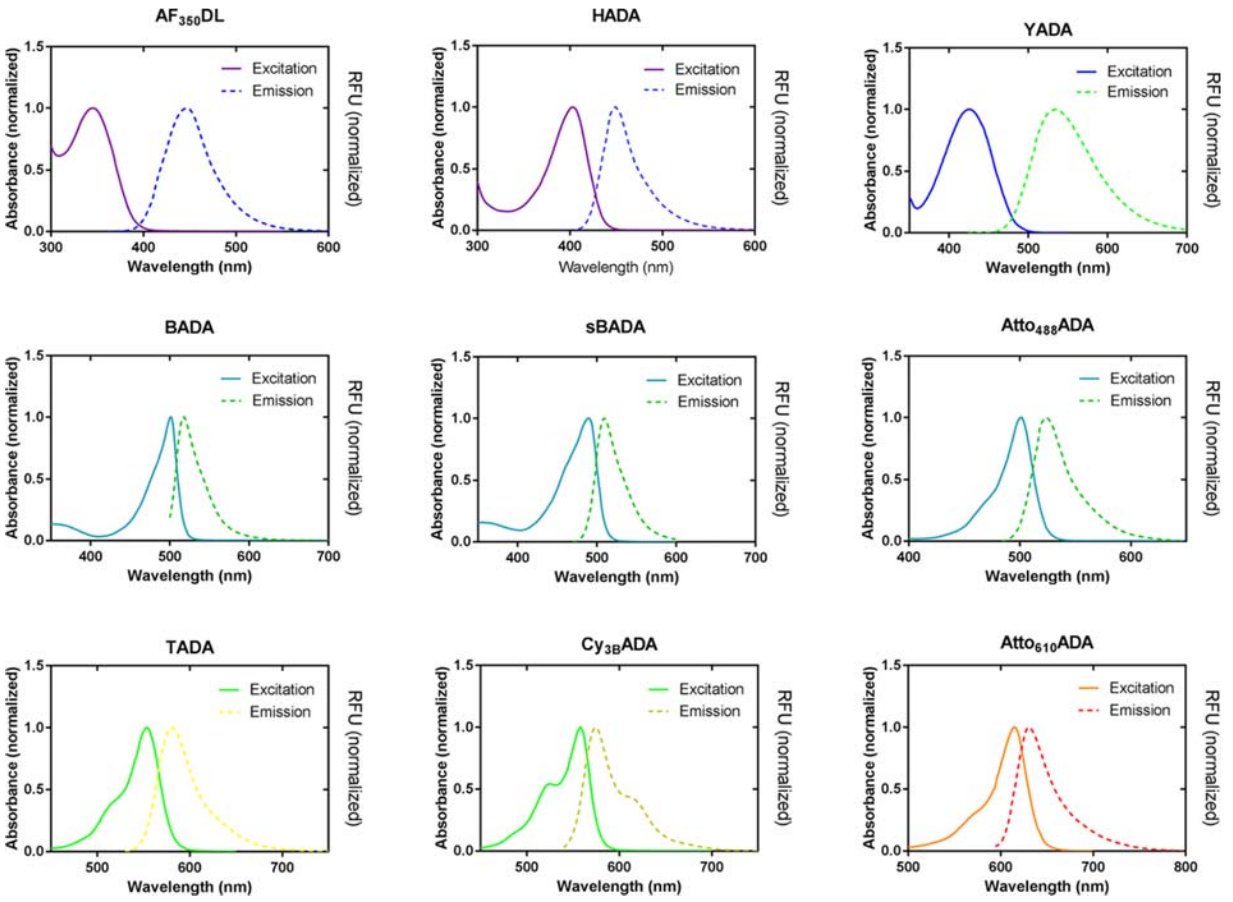

Figure S1: Excitation (solid lines) and emission (dashed lines) spectra of FDAAs.

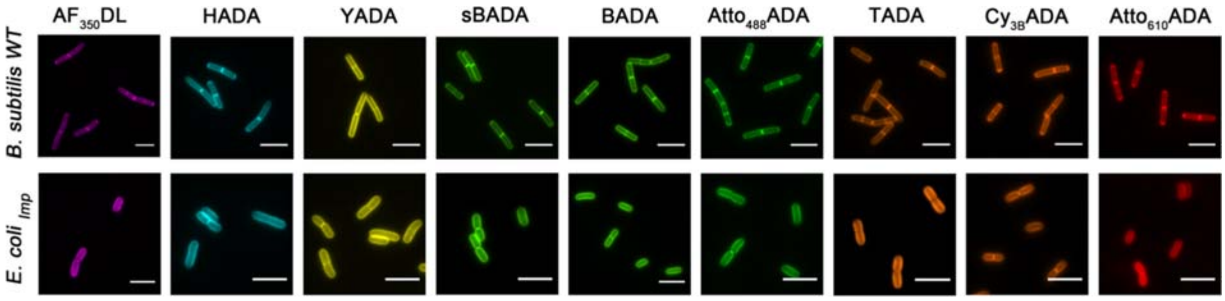

Figure S2: FDAA labeling of *B. subtilis* and *E. coli imp4213*. Cells were labeled with FDAAs for 1 h at a concentration of 0.5 or 1 mM. Microscopy settings (light source power, exposure, EM gain, ex/em filters) were adjusted individually for each FDAA to optimize the signal-to-background ratio. Scale bar: 5 µm.

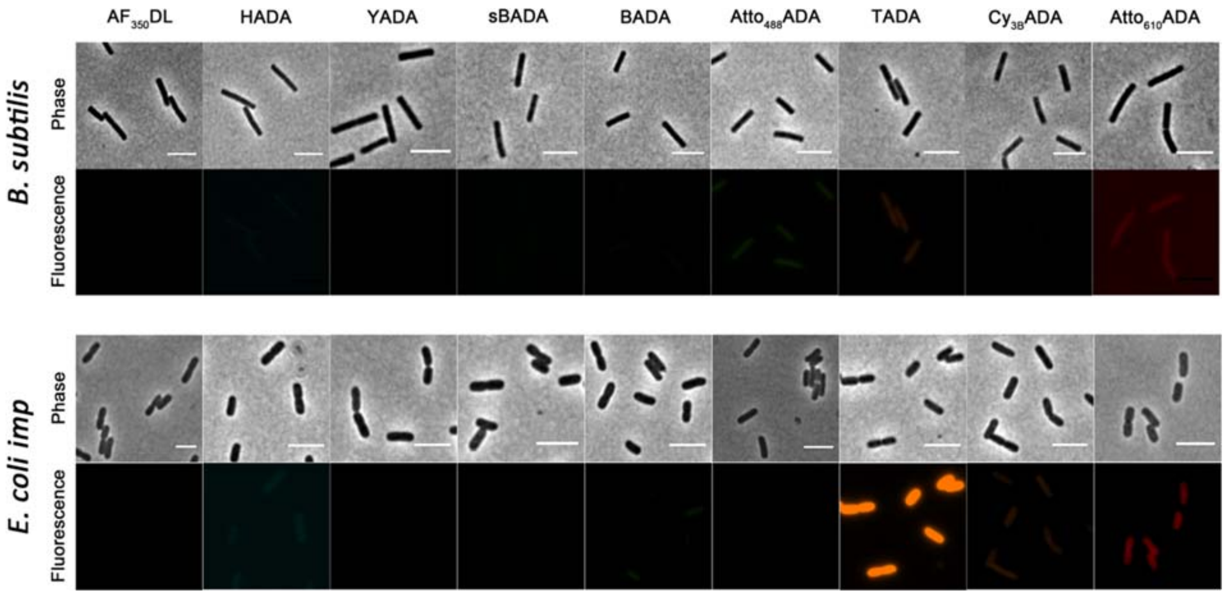

Figure S3: FDAA labeling of pre-fixed *B. subtilis* and *E. coli imp4213* cells. Cells were fixed with 70% ethanol (1 h, ice bath) followed by FDAA incubation for 1 h. The labeling protocol, microscopy settings, and image processing conditions are the same as in Fig. S2. Scale bar: 5 μm. Strong, non-specific signal of TADA was found in pre-fixed *E. coli*. This signal might result from TADA being trapped inside the cells after fixation which changes outer-membrane permeability toward the probe. *E. coli* labeling using TALA (the L-isomer of TADA) without fixation showed no labeling signal, indicating that TADA in live cells labels peptidoglycan specifically (data not shown).

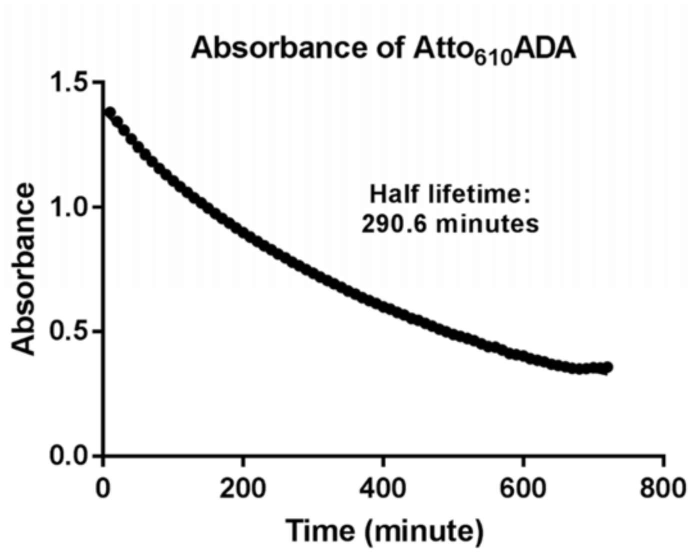

Figure S4: Atto$_{610}$ADA thermo-stability. Time-lapse monitoring of Atto$_{610}$ADA absorbance in pH 7.4 PBS at 37 °C for 12 h. Measurement interval: 10 min. The half-life was calculated using a fit to a decaying exponential.

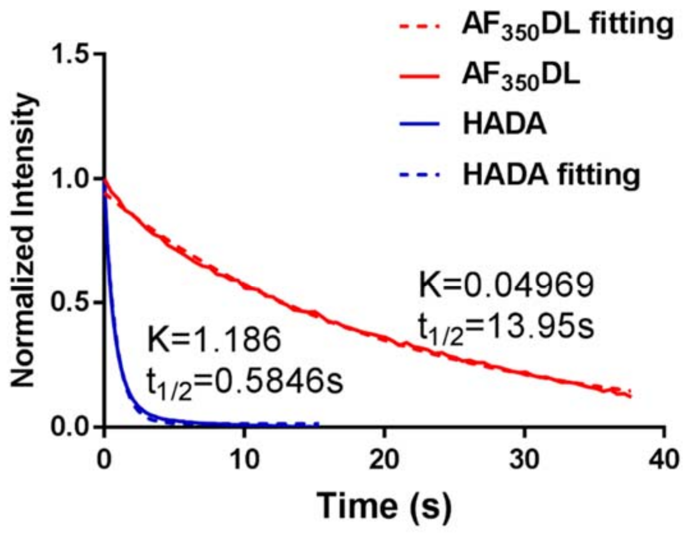

Figure S5: Comparison of photo-stability between HADA and $AF_{350}DL$. The half-life ($t_{1/2}$) and exponential decay coefficient (EDC, K) was calculated using fitting to exponential decay in Prism.

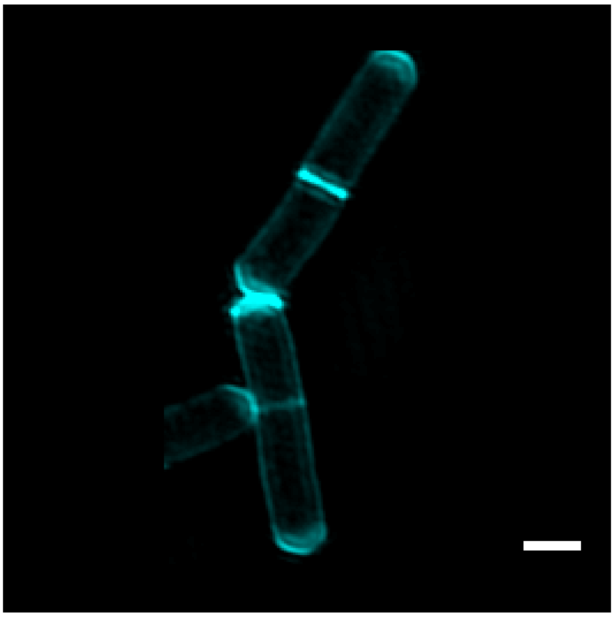

Figure S6: 3D-SIM image of *B. subtilis* labeled by 1 mM HADA for 1 h. Scale bar: 1 μm. 3D-SIM images were collected on a DeltaVision OMX system (Applied Precision Inc., Issaquah, USA) equipped with a 1.4NA Olympus 100X oil objective. A 405-nm laser source and 419-465-nm emission filter were used for collecting HADA signal. The *z*-axis scanning depth was 3 μm. Immersion oil with refraction index of 1.514 was used. SIM images were reconstructed using softWoRx.

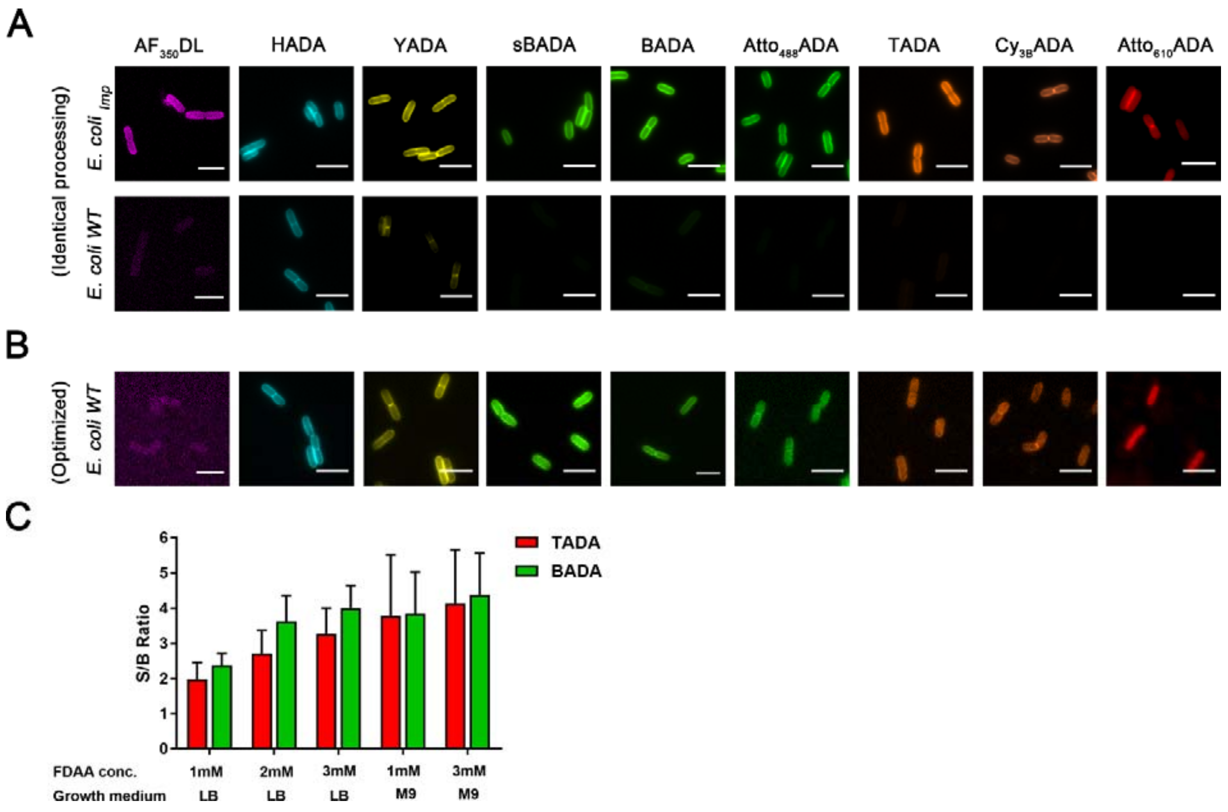

Figure S7: FDAA labeling of *E. coli*. A) Comparison of FDAA labeling efficiencies in *E. coli imp4213* BW25113 cells (top) and wild-type *E. coli* BW25113 (bottom). Cells were labeled with various FDAAs for 1 h. Images were acquired and processed under the same conditions between the two strains. B) Wild-type *E. coli* BW25113 images from the same data set in (A) with optimized processing conditions. Scale bar: 5 µm. C) S/B ratio of PG labeling with different FDAA concentration and culture medium.

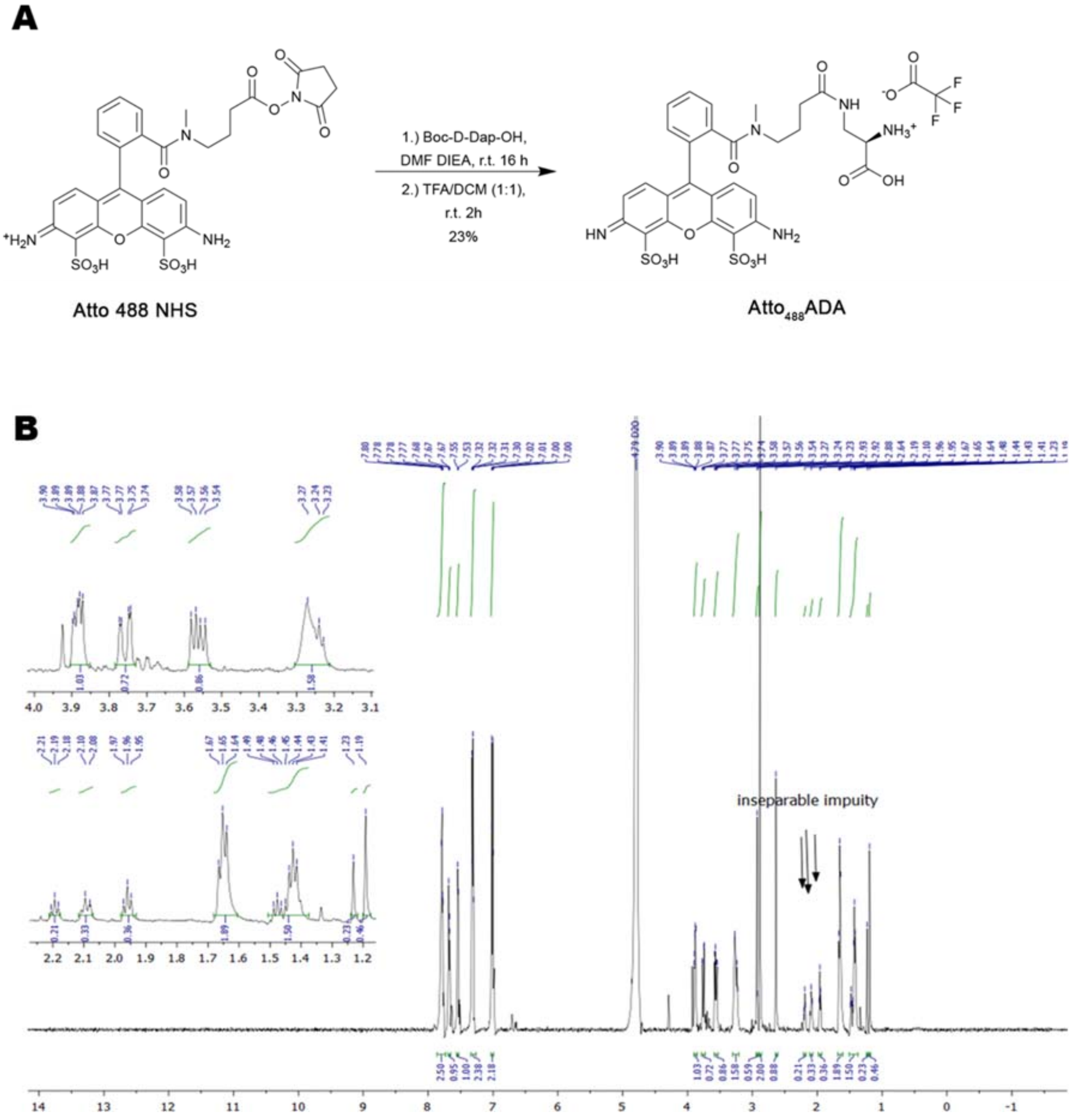

Figure S8: Synthesis scheme (A) and ^{1}H NMR (B) of Atto$_{488}$ADA.

A

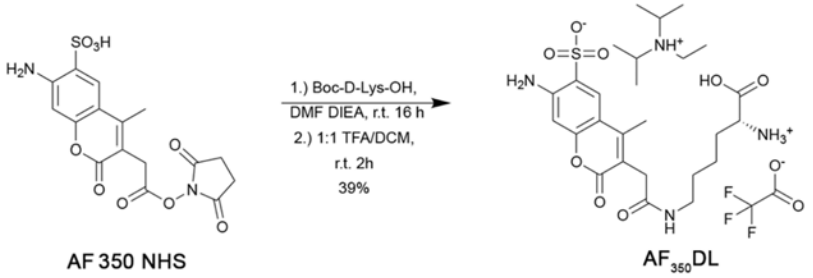

B

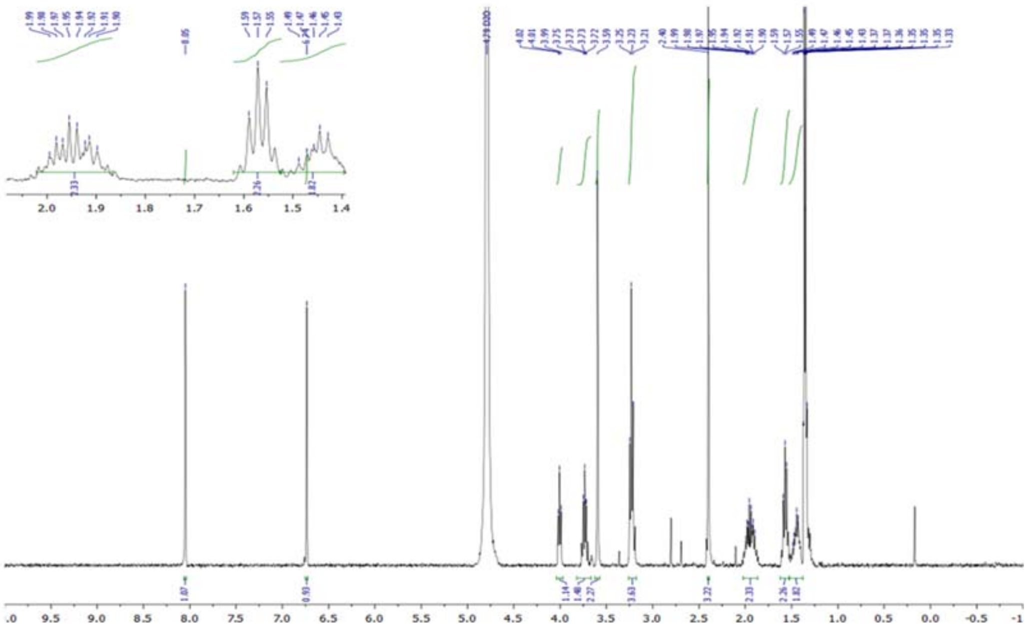

Figure S9: Synthesis scheme (A) and ^{1}H NMR (B) of Atto$_{350}$DL.

A

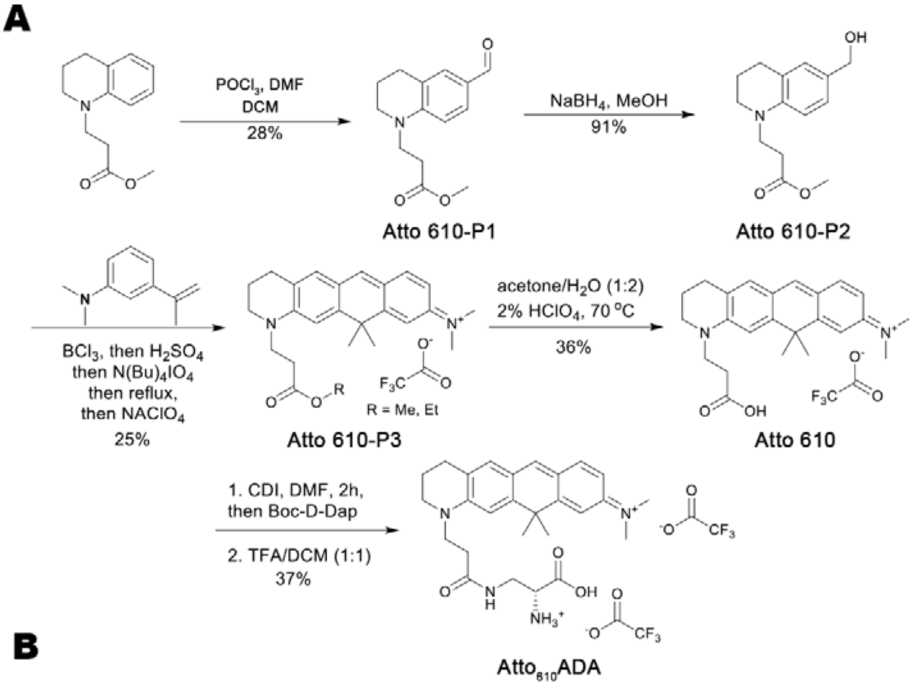

B

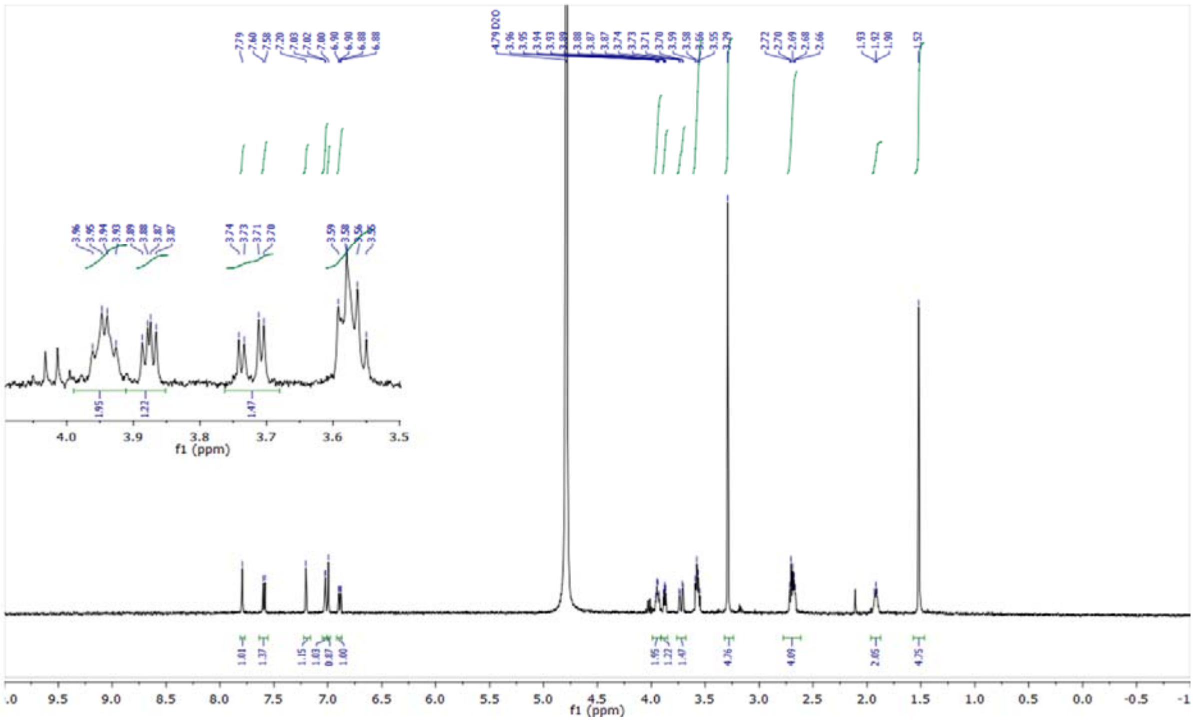

Figure S10: Synthesis scheme (A) and ^{1}H NMR (B) of Atto$_{610}$ADA.

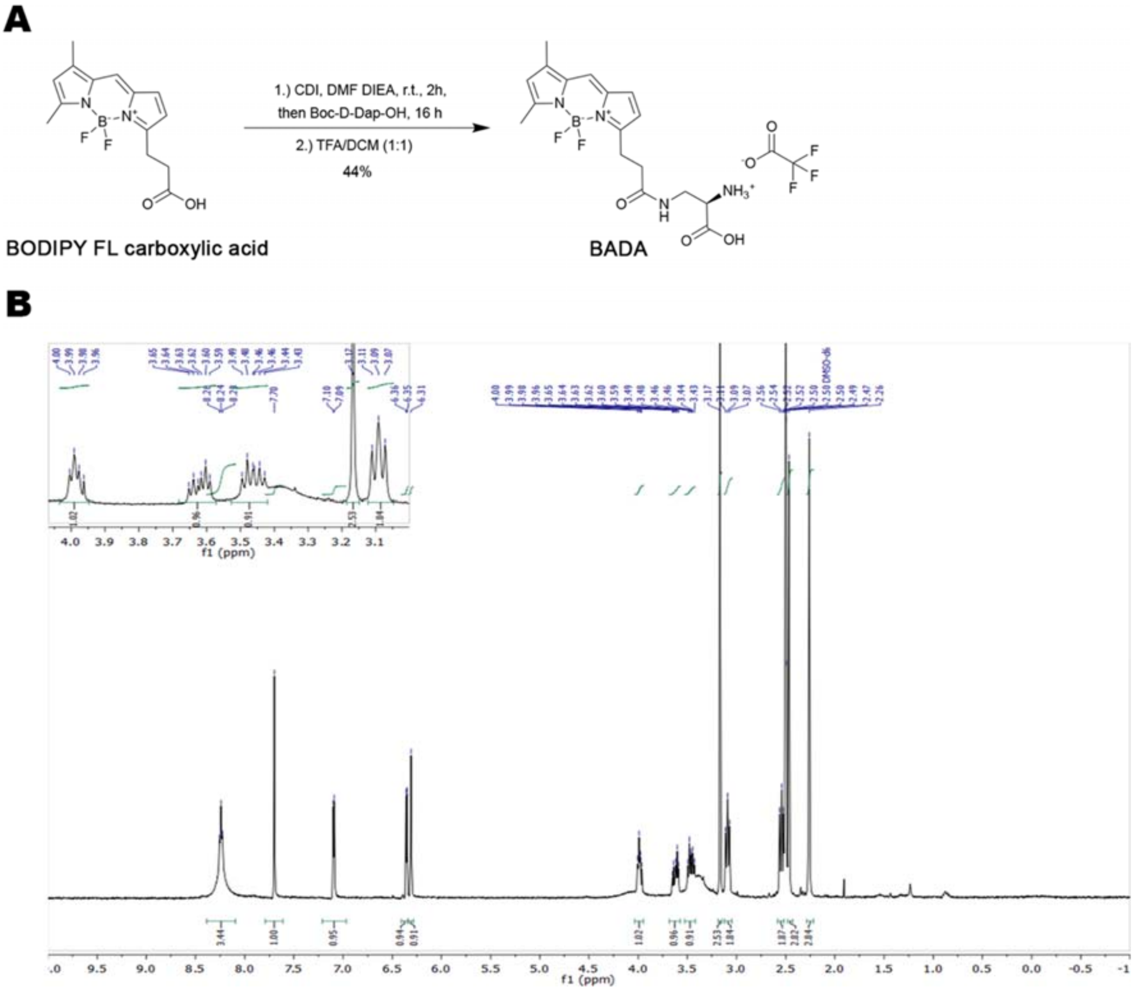

Figure S11: Synthesis scheme (A) and ^{1}H NMR (B) of BADA.

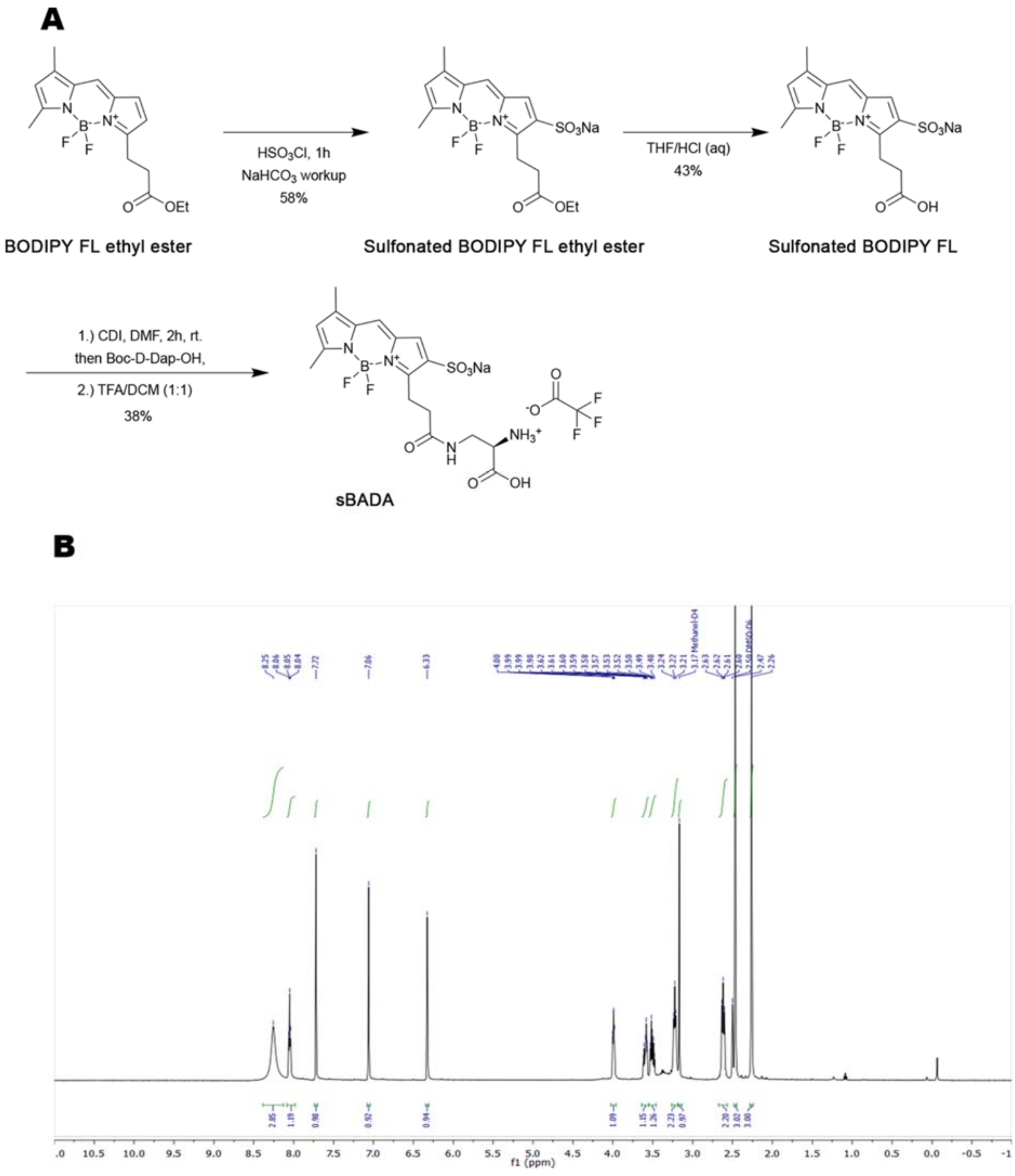

Figure S12: Synthesis scheme (A) and ^{1}H NMR (B) of sBADA.

A

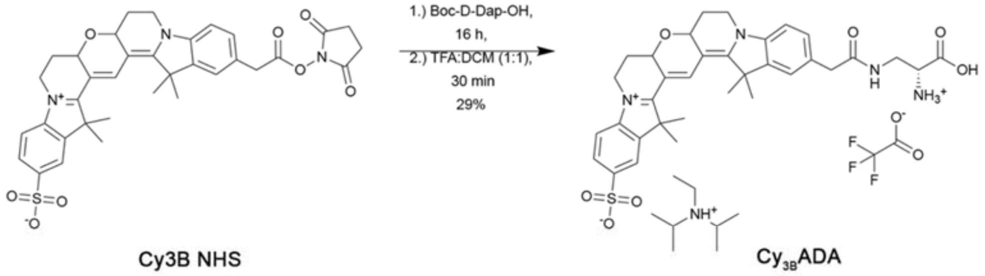

B

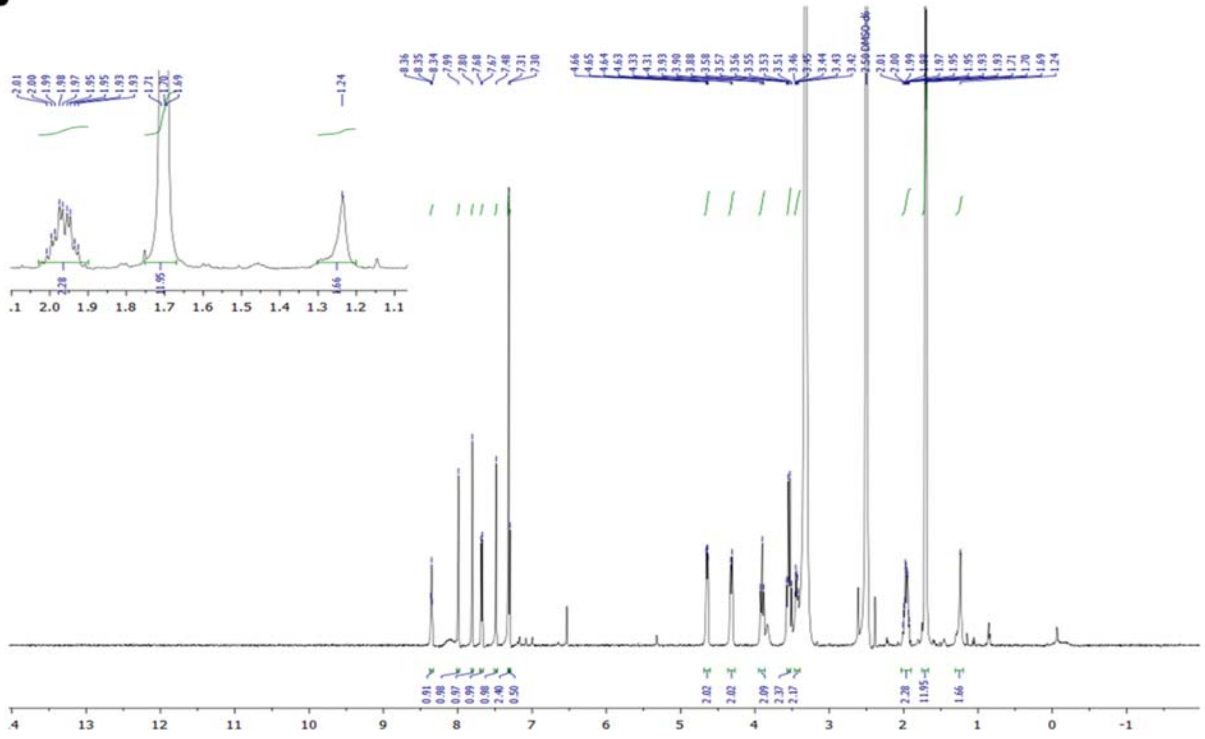

Figure S13: Synthesis scheme (A) and ^{1}H NMR (B) of Cy$_{3B}$ADA.

A

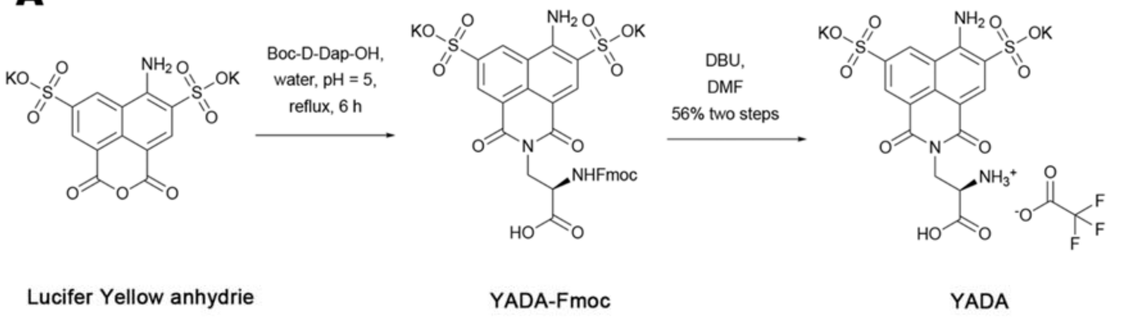

B

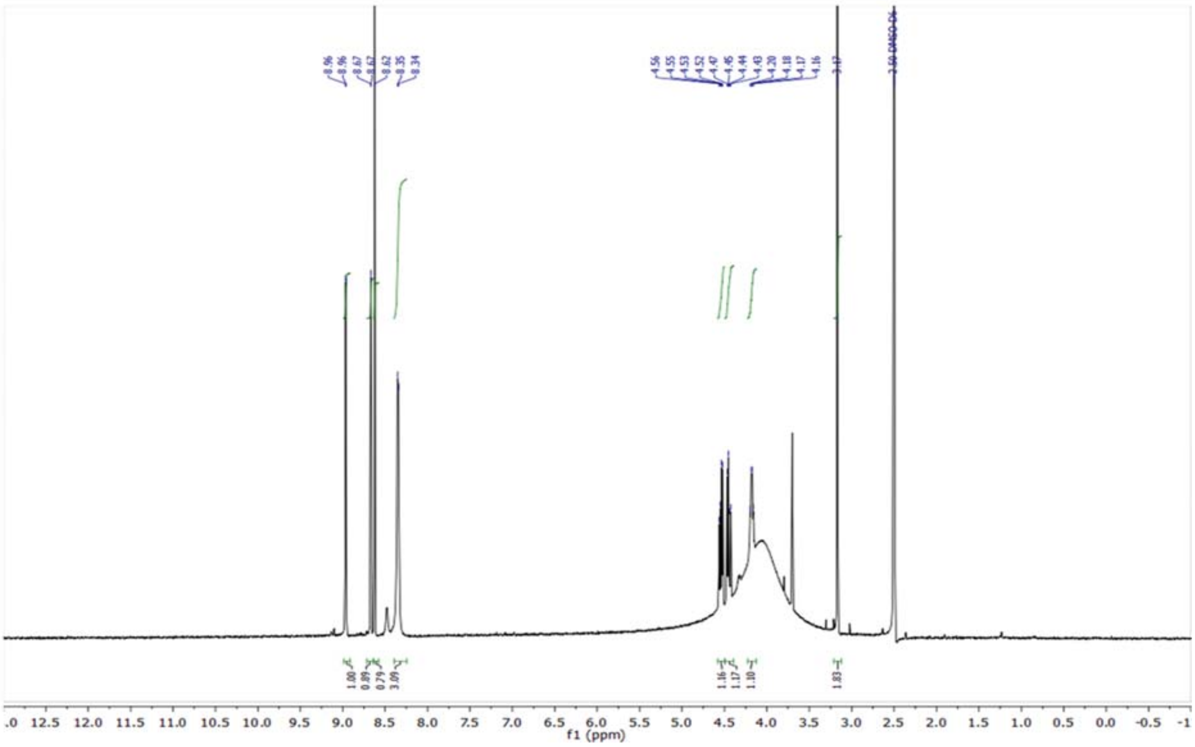

Figure S14: Synthesis scheme (A) and ^{1}H NMR (B) of YADA.

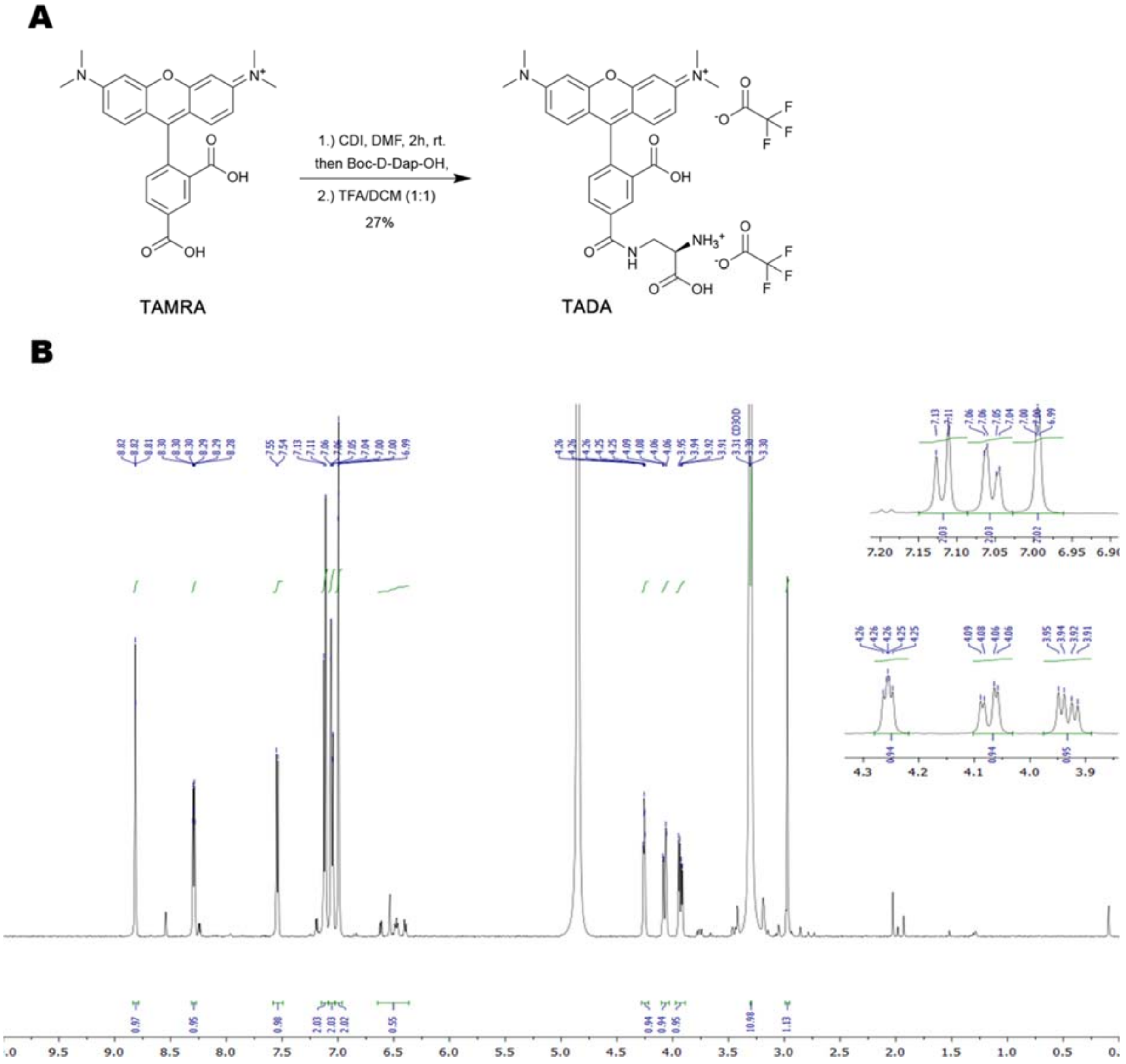

Figure S15: Synthesis scheme (A) and ^{1}H NMR (B) of TADA.

Dye (Emission)	Water-Solubility ($LogD_{7.4}$)[a]	Photo-stability (Exponential decay coefficient)	Labeling efficiency *E. coli* BW25113 (Signal-to-background ratio)
Violet-to-Blue			
$AF_{350}DL$	< -2	0.04969	1.1 ± 0.02
HADA	-1.059 ± 0.076	1.186	6.6 ± 0.38
**Green**			
sBADA	< -2	0.3842	2.0 ± 0.09
BADA	0.28 ± 0.088	0.4578	3.0 ± 0.15
$Atto_{488}ADA$	-1.96 ± 0.125	0.05695	1.4 ± 0.15
**Green-to-Yellow**			
YADA	-1.733 ± 0.389	0.03538	4.0 ± 0.18
**Yellow-to-Orange**			
TADA	< -2	0.0276	1.5 ± 0.02
$Cy_{3B}ADA$	-1.69 ± 0.3	0.133	1.3 ± 0.04
**Red**			
$Atto_{610}ADA$	-0.23 ± 0.185	0.0043	2.1 ± 0.32

Table S1: FDAA characterization. This table summarizes the raw values of FDAA water-solubility ($LogD_{7.4}$), photo-stability and labeling efficiency in *E. coli* BW25113. [a] Due to the detection limit of the plate reader, $LogD_{7.4}$ values less than -2 show high standard deviation. Those values are assigned "<-2" in the table.

Species	Background	Source	#	Gram	Media	T (°C)
Bacillus subtilis (WT)	3610	Brun Lab (IUB)	YB7447	+	LB	37
Streptomyces venezuelae (WT)		Brun Lab (IUB)	YB6837	+	LB	30
Escherichia coli (WT)	BW25113	Brun Lab (IUB)	YB7421	-	LB	37
Escherichia coli imp4213	BW25113	Huang Lab (Stanford)	KC440	-	LB	37
Escherichia coli imp4213 Δ6 Ldt	BW25113	Huang Lab (Stanford)	KC441	-	LB	37
Lactococcus lactis (WT)		Brun Lab (IUB)	YB7412	+	LB	30

Table S2: Bacterial species used in this study.

Name	Application	Ex Range (nm)	Em Range (nm)	FDAAs
Chroma 395/25	Excitation (DAPI)	382.5-408		$AF_{350}DL$, HADA, YADA
Chroma 470/24	Excitation(GFP/FITC)	458-482		BADA, sBADA, $Atto_{488}ADA$
Chroma 550/15	Excitation (Cy3)	542.5-558		TADA, $Cy_{3B}ADA$
Chroma 640/30	Excitation (Cy5)	625-655		$Atto_{610}ADA$
Chroma 435/26	Emission (DAPI)		422-448	$AF_{350}DL$, HADA
Chroma 510/40	Emission (GFP/FITC)		490-530	BADA, sBADA, $Atto_{488}ADA$, YADA
Chroma 595/40	Emission (Cy3)		575-615	TADA, $Cy_{3B}ADA$
Chroma 705/72	Emission (Cy3)		669-741	$Atto_{610}ADA$

Table S3: Microscope filter sets for excitation and emission.

Author contributions

FDAA synthesis by YH, JR, JY, EP, ST, BM and EH. FDAA property characterization by YH. FDAA labeling and imaging by YH and EK. STORM data by EK, ECG, TKL, and KCH. Manuscript drafting by YH, JR, EK, KCH, YVB, and MSV. All authors were involved in the design of experiments in the paper.

References

1. Arden-Jacob *et al.*, New fluorescent markers for the red region. *Spectrochim. Acta Part A Mol. Biomol. Spectrosc*. 2001, **57**, 2271-2283.
2. Loudet *et al.*, BODIPY Dyes and Their Derivatives: Synthesis and Spectroscopic Properties. *Chem. Rev.*, 2007, **107**, 4891-4932.
3. Kuru *et al.,* In Situ Probing of Newly Synthesized Peptidoglycan in Live Bacteira with Fluprescent D-Amino Acids, *Angew. Chem. Int. Ed*., 2012, **51**, 12519-12523.
4. Joshua V *et al.*, Ultra-bright Photoactivatable Fluorophores Created by Reductive Caging. *Nat. Methods*, 2012, **9**, 1181-1184.
5. Huang *et al*., Three-Dimensional Super-Resolution Imaging by Stochastic Optical Reconstruction Microscopy. *Science*, 2008, 319, 810-813
6. Stuurman *et al*., Computer control of Microscopes using µManager. *Curr. Protoc. Mol. Biol.,* 2010. DOI: 10.1002/0471142727
7. Andres A *et al*, Setup and validation of shake-flask procedures for the determination of partition coefficients (logD) from low drug amounts., *Eur J Pham Sci.*, 2015, **76**, 181-191.
8. Adrien D *et al.*, MicrobeJ, a tool for high throughput bacterial cell detection and quantitative analysis. Nature Microbiology, 2016, **1**, 1.
